# HybridBranchNetV2: Towards reliable artificial intelligence in image classification using reinforcement learning

**DOI:** 10.1371/journal.pone.0314393

**Published:** 2025-02-10

**Authors:** Ebrahim Parcham, Mansoor Fateh, Vahid Abolghasemi

**Affiliations:** 1 Faculty of Computer Engineering, Shahrood University of Technology, Shahrood, Iran; 2 School of Computer Science and Electronic Engineering, University of Essex, Colchester, United Kingdom; Khalifa University of Science and Technology, UNITED ARAB EMIRATES

## Abstract

Many artificial intelligence (AI) algorithms struggle to adapt effectively in dynamic real-world scenarios, such as complex classification tasks and object relationship extraction, due to their predictable but non-adaptive behavior. This paper introduces HybridBranchNetV2, an optimized hybrid architecture designed to address these challenges. The key novelty of our approach lies in the integration of reinforcement learning for adaptive feature extraction and the use of graph-based techniques to analyze object relationships in complex environments. By dynamically adjusting feature extraction based on feedback from the environment, the model improves adaptability, while graph-based methods allow for a more comprehensive analysis of object relationships. Our extensive evaluations demonstrate that HybridBranchNetV2 achieves average 91.75% accuracy over four different challenging datasets. In particular, a 14% improvement obtained on the Visual Genome dataset and ImageNet 1K compared to the original HybridBranchNet model. Additional testing on CIFAR, Flowers, and ImageNet datasets revealed improvements of 6%, 1%, and 6%, respectively. These advancements not only enhance classification accuracy but also ensure efficient computation, making HybridBranchNetV2 suitable for real-time applications with minimal risk of overfitting. The proposed framework demonstrates significant improvements in adaptability, performance, and computational efficiency, addressing critical limitations in current AI models.

## I. Introduction

Artificial Intelligence (AI) denotes the evolution of computer systems capable of performing tasks conventionally requiring human intelligence. This includes capabilities such as speech recognition, decision-making, and problem-solving [1, 2]. The significant advancement in AI research has been the inception of Deep Neural Networks (DNNs). DNNs are modeled on the structure and function of the human brain, consisting of multiple layers of interconnected nodes. Each node processes data, relaying it to successive layers, enabling DNNs to discern patterns in substantial datasets. This capability is crucial in applications ranging from image and speech recognition to natural language processing and self-driving vehicles [3].

Nevertheless, DNN training poses significant challenges. The demand for extensive labeled data–data annotated with specific attributes–stands paramount. Acquiring and annotating of such datasets can be both time-consuming and expensive, especially in sectors like healthcare or finance where data confidentiality is paramount [4]. Overfitting, a scenario where the model intricately adapts to training data but falters with novel data, remains a salient challenge. Furthermore, DNNs are susceptible to adversarial attacks, compromising the reliability of their decisions, especially in security-critical applications [5]. Beyond these technical challenges, Studies indicate that DNNs can confidently predict outcomes for images that are indistinguishable from humans, demonstrating vulnerability to misclassification. The findings underscore the potential for misleading decisions and emphasize the need for ethical considerations in the deployment of deep learning technologies [6]. Nevertheless, the potential of DNNs remains vast. Efforts persist to counteract these challenges, to craft more resilient, transparent, and ethically sound AI systems [7].

Deep neural networks have significant potential for innovation in the field of artificial intelligence, but their deployment and application come with numerous challenges [8]. These challenges include issues such as data quality and accuracy, the ability of models to generalize, security vulnerabilities, transparency in decision-making, and ethical considerations. Current research in this field is focused on addressing these challenges to ensure that artificial intelligence systems not only contribute to societal progress but also minimize the risks associated with them. Furthermore, academic experiences in the field of deep learning, which include university research and practical applications in this area, have shown us that using limited input data in deep neural networks can have its limitations. These limitations become particularly apparent when neural networks must deal with environments and conditions that are variable and complex. In other words, when the input data used for training and tuning neural networks are not sufficiently diverse and extensive, these networks may struggle with generalizing findings and making accurate predictions in new and unknown conditions. This issue becomes especially important in cases where neural networks must be capable of recognizing patterns or making complex decisions in data with unusual or rare structures. For example, in situations where the network must recognize images with features very different from the training data or make predictions based on data with temporal or spatial variations, having diverse and comprehensive input data is of great importance.

This article seeks to augment neural network models by fusing textual and visual features and creating the necessary diversity in the input data. The diversity is achieved by combining different types of textual and visual features. This combination aims to enrich the neural network’s learning experience, allowing it to recognize and understand more complex patterns and scenarios than it could with a more homogeneous or limited dataset. In this paper, we address the relationship between descriptive information of each image and the improvement in image classification accuracy. For this purpose, we augment our base image classification model (HybridBranchNet) by text interpretation blocks relying on descriptive graphs. We found that visual information provides limited knowledge about images. Therefore, by integrating text and images using a graph-based technique, we presented a new model that is not complex and does not suffer from language model errors. Previous methods for integrating text and image relied on language models, which significantly increased model complexity and introduced language model errors. In these methods, image feature extraction is dependent on language model interpretation. Additionally, network errors and imperfections are normally transferred to the feature extraction part. In the proposed method, by using textual graphic patterns, feature extraction becomes completely dependent on objects and their relationships. This characteristic eliminates language model errors in the feature extraction stage. Our experiments show that the image classification accuracy of the proposed model is increased compared to the methods based on the language model. Furthermore, accuracy on unseen images has been improved compared to our original HybridBranchNet model.

To further clarify the scope of this work, we provide an example here: A neural network might struggle to detect a small cat in a large, complex scene like a five-lane road using only visual data. However, by introducing additional types of data, such as textual descriptions or tags that might provide context (like the presence of a cat in the image), the neural network can gain a more comprehensive understanding of the scene. Such data enrichments, with its requisite diversity, enables the neural network to make more accurate predictions or identifications, even in complex or challenging scenarios. This research proposes a solution that involves creating a supplementary model to enhance the recognition capability of the main model, which identifies the object with textual descriptions.

To fully leverage the features of textual content, we convert texts into graphs. Graphs can always serve as the best tools for describing and illustrating relationships. This method provides the ability to display connections more accurately and clearly. Additionally, by increasing or decreasing these connections in the graph, we can extract new meanings.

Our methodology starts by transforming a generic image into a descriptive graph, meticulously detailing each object, their attributes, and interrelationships. This structure significantly enhances our ability to understand multifaceted images. In the proposed architecture, we integrate both textual and visual elements. This approach builds upon the foundational architecture referenced in [9], which we will describe in more detail in Section II. The base architecture has significantly reduced the number of parameters, yet it has enhanced efficiency and effectiveness. This model not only demonstrates exceptional data extraction capabilities but also maintains a balance between nimbleness and efficiency, making it well-suited for a variety of platforms. In summary, the key contributions of this research include:

- **Reinforcement learning for feature extraction:** Our study introduces a novel approach by incorporating reinforcement learning techniques into the feature extraction process. While traditional methods often rely on supervised or unsupervised learning for feature extraction, our approach harnesses the power of reinforcement learning to dynamically adapt feature extraction based on feedback from the environment. This enhances not only the adaptability of the feature extraction process but also enables the system to learn optimal feature representations tailored to specific tasks or domains.
- **Graph features for analyzing object relationships:** Another key innovation of our work lies in the utilization of graph features for analyzing relationships between objects. Unlike conventional approaches that mainly focus on individual object features, our method considers the relational context by representing objects and their interactions as nodes and edges in a graph structure. By leveraging graph-based techniques, we enable more comprehensive analysis of object relationships, facilitating tasks such as object recognition, classification, and localization in complex scenes or datasets.

In the initial version of the HybridBranchNet method, one of its fundamental challenges was the reliance on only one loss function for object classification. This limitation reduced the network’s ability to gain a more comprehensive understanding of the image data. Specifically, the network was primarily focused on classifying data and lacked the ability to extract more features from the image. This issue led the research team to move away from traditional structures and design a novel hybrid approach that could extract more information from the image and provide stronger feature representations. This broader information includes object detection and relationships between objects that might exist in the image, beyond just the dominant class. To develop a network capable of interactively and continuously extracting relevant features while maintaining fewer parameters, the design of an interactive network was deemed necessary.

In this regard, reinforcement learning was chosen as a suitable solution. One of the main reasons for selecting reinforcement learning in this model is its ability to extract new and hidden features from images. This process allows the network to converge more effectively by considering the rewards and penalties it receives during learning, dynamically optimizing as it progresses. In other words, reinforcement learning helps the network select the best possible features at each stage of learning and gradually extract more information from the image. This capability is especially beneficial for complex image data, as it enables the model to gain a deeper understanding of the image content.

Another key advantage of this approach is the reduction in the number of trainable parameters in the network. Complex models with many parameters not only consume more computational resources but can also face challenges like excessive complexity and overfitting. By adopting reinforcement learning and implementing it within the HybridBranchNetV2 architecture, the research team was able to maximize the use of image data with a minimal number of network parameters. This approach not only reduced the trainable parameters but also led the network towards a more natural and smoother convergence.

Ultimately, this design resulted in improved accuracy in image classification, as the network was able to extract more information from the image and optimally apply it in object classification. Therefore, the main goal of designing HybridBranchNetV2 was to reduce the number of trainable parameters, extract new features, and add more information from the image to the network, which ultimately led to improved model accuracy.

The rest of the paper is structured as follows: Section II offers a literature review highlighting pivotal AI advancements. Section III delineates our proposed methodology. Section IV presents detailed experimental insights. Section V sketches a discussion on the obtained results and achievements. Finally, Section VI provides concluding remarks and future research avenues.

## II. Related works

In this section, we offer a summary of recent literature in artificial intelligence, particularly emphasizing advancements and research in deep learning and image classification techniques. This overview highlights the cutting-edge methodologies and innovative approaches being explored to push the boundaries of accuracy and efficiency in image recognition and analysis, focusing on the technical aspects and developments within these areas. Our discussion highlights their methodologies and findings, emphasizing the critical role of input data in deep learning and machine learning. We also investigate how integrating auxiliary components into our architecture can reduce this dependency, moving towards more reliable AI systems. According to the challenges discussed above, we categorize the related AI models into three distinct categories which are described as follows.

Each of these categories include a range of topics and issues that are crucial to the development and application of AI and deep learning technologies. Organizing the challenges into these categories aims to create a more coherent and comprehensive understanding of the current state of AI research and its future directions.

### II.I. Advances and challenges in deep learning

In [10], the limitations of deep learning algorithms in adversarial environments are discussed, where an attacker intentionally manipulates data to deceive the algorithm. Nicolas, the author, argues that deep learning algorithms lack robustness against adversarial attacks, which can have serious consequences in applications such as autonomous vehicles and cybersecurity. Nicolas discusses potential solutions and outlines future directions for addressing these limitations. Drawing insights from [10], we can infer those errors or inaccuracies in the network’s inputs, such as precise graph or image requirements, may not necessarily result in substantial errors in the overall performance of the network.

The paper [11] explores strategies for effectively training deep neural networks with limited labeled data, overcoming labeling constraints. The authors explore challenges such as data availability, high labeling costs, and data ambiguities. To address these challenges, their proposed solutions include transfer learning, AI-driven data augmentation, and the integration of multiple data sources. These methods aim to alleviate labeling constraints and enhance the performance of deep learning.

In [12], an effective semi-supervised learning approach, termed Augmented Distribution Alignment, is introduced. The authors identify a sampling bias in semi-supervised learning, arising from limited labeled samples and resulting in a distribution mismatch between labeled and unlabeled data. They propose aligning these distributions to improve learning outcomes. The paper [13] unveils a novel technique to enhance the scene graph generation (SGG) performance. SGG is a task dedicated to extracting (subject, predicate, object) triplets from images. The proposed method in [13], named IETrans, leverages both internal and external data transfer to boost the quality and quantity of SGG data. Internal data transfer involves sharing information from the object detection branch to the relation prediction branch within the SGG model. In contrast, external data transfer involves incorporating knowledge from a vast pre-trained language model into the SGG model. The article demonstrates that IETrans can achieve exceptional results on multiple benchmarks, such as Visual Genome and GQA [14, 15].

In [16], a modified least squares regression model is introduced that incorporates two constraints for improving classification accuracy. These constraints aim to minimize the variance within image classes and maximize the separation between different classes. The method enhances the model’s capability to distinguish between high-dimensional image data, outperforming traditional regression models. It utilizes an optimization algorithm tailored for this double-constrained setup, applicable across various image recognition scenarios. In [17], a novel method for classifying defective images using deep neural networks (DNN) is introduced. By optimizing the neural network on both defective and non-defective images, the proposed approach significantly improves the accuracy of image classification. The results demonstrate a noticeable increase in classification accuracy through neural network optimization on defective images. In conclusion, this method effectively enhances image classification precision by detecting and distinguishing defective images.

In [18], an innovative approach to content-based image retrieval (CBIR) is proposed, introducing a query-sensitive co-attention mechanism for large-scale tasks. In contrast to traditional methods, the proposed method dynamically adapts to query features, thereby enhancing retrieval performance. To reduce computational costs, the method incorporates clustering of selected local features. Experimental results demonstrate the effectiveness of the co-attention maps, especially in challenging scenarios with significant variations in how images are taken between the query and its matching image.

In [19], an innovative approach is introduced to address the challenge of Unsupervised Domain Adaptation (UDA) in image classification. Unlike conventional methods, the proposed method focuses on learning a unified classifier for both the source and target domains, eliminating the need for explicit domain alignment.

In [20], the Reverse Contrastive Learning (RCL) approach is introduced for high-quality and diverse image generation in few-shot settings. RCL utilizes a unique regularization method based on the correlation between generated samples, demonstrating superiority over existing State-Of-The-Art (SOTA) methods in few-shot scenarios and remaining competitive in low-shot settings.

In [21], challenges in deep generative models for multi-modal data were noted. Introducing a novel conditional multi-modal discriminative model, the research maximizes mutual information between joint representations and missing modalities. The proposed model achieves state-of-the-art results in downstream tasks such as classification and image generation.

### II.II. Image classification

In the field of image classification, B. Hanin explores the robustness of deep learning models to label noise in large datasets [22]. The author argues that deep learning models can perform well even in the presence of significant noise in training data labels. This argument challenges the traditional belief that noisy labels can severely impact the performance of machine learning algorithms. The article back this claim with theoretical analysis and empirical evidence, suggesting that the robustness to label noise may stem from the over-parameterization of deep learning models. The discoveries in this article could carry significant implications for the development and deployment of deep learning models in real-world applications [22].

In [23], a novel structural regularized semi-supervised learning model is introduced. This model, specifically designed for Multiview data, is named Adaptive Multiview Semi-supervised model (AMUSE). The model takes advantage of a priori graph structure to learn weights, which is considered more reasonable compared to weight regularization. Semi-supervised learning models for Multiview data are considered significant in image classification tasks due to their ability to easily obtain heterogeneous features, making them both economical and effective.

In [24], a novel multi-stage approach for image scene classification is presented. The approach utilizes high-level semantic features extracted from the image content. In the initial stage, the identification of object boundaries within the images are carried out, laying the groundwork for further analysis. The article highlights the importance of image scene classification, emphasizing both the challenges and significance of utilizing low/high-level features for this task. The high-level features, grounded on semantic concepts, provide a more accurate and nuanced model that closely reflects the human perception of the image scene content.

In [25], a representative sampling model (CALR) for active learning is introduced. This model selects valuable samples without requiring an initial labeled set or iterative feedback from target models. To efficiently address the cold-start problem, this model leverages self-supervised learning, clustering, and manifold learning to identify informative images for labeling from the start. These strategies are designed to initiate classification on large unlabeled datasets, especially adept at handling data imbalance, and overcoming the limitations of random selection. The paper introduces a structured framework for initiating the training of classification models in scenarios with unavailable labeled data. This ensures a balanced representation of classes and enhances the efficiency of the learning process.

In [26], a deep learning model for image classification and object detection in waste management, combining CNN and LSTM networks with pre-trained ImageNet weights is proposed. This hybrid approach improves accuracy by addressing complexity and data limitations, outperforming EfficientNet models. Grad-CAM visualizations show precise object focus, and the method supports low-power device deployment, making it suitable for practical applications.

In [27] a hybrid CNN-LSTM model for image classification and object detection, focusing on waste management. Using transfer learning with ResNet-50 and LSTM, it classifies waste into recyclable and organic categories. The model achieves high accuracy with fewer parameters, outperforming EfficientNet models, and is suitable for low-power devices.

In [28], a similarity-based method is introduced, creating a new class of methods for multi-label learning. This method is generalized in the paper, establishing a new framework for classification tasks. The proposed framework aims to achieve promising performance in both multi-label learning and classification tasks.

The study [9] explores systematic examination of branch network structures within ConvNet deep neural networks, proposing a novel architecture known as HybridBranchNet. These networks generally follow a consistent architecture that can be scaled and adjusted for various applications. Increasing the network dimensions—like depth, resolution, and width—leads to more trainable parameters and improved accuracy. However, it also contributes to network complexity. To strike a balance speed, reduce network size, and optimize accuracy, the study introduces a novel scaling method. This method optimizes depth, width, and resolution dimensions based on branch neural networks. This approach gives rise to a family of HybridBranchNet networks that outperform traditional ConvNets. For example, HybridBranchNet3 achieves an 83.1% classification accuracy [9]. In addition, there are other approaches such as the CoAtNet architecture, which seamlessly blends convolution and attention mechanisms to efficiently handle data of varying sizes. By integrating both convolutional and attention-based operations, CoAtNet enhances the flexibility and scalability of neural networks. CoAtNet is designed to address challenges linked with processing data of diverse sizes, marking it as a promising strategy for numerous applications.

The study [30] investigates a novel geometric-spatial image representation for scene classification. The method combines geometric and histogram features to detect and classify scenes. By integrating these two types of features, the method provides a more precise and comprehensive representation of scenes, leading to higher accuracy in their classification.

In [31, 32] the Vision Transformer (ViT) architecture is used by introducing factorized attention to better handle large-scale image classification tasks like ImageNet. The method achieves state-of-the-art performance, outperforming traditional convolutional networks in terms of both accuracy and training efficiency. The authors discuss how scaling transformers can help to further optimize deep learning models for visual tasks.

In [33] an approach for improving Vision Transformer training is proposed by token labeling, which helps the model learn finer-grained features from images. This method has been evaluated on the ImageNet dataset and demonstrates superior performance compared to previous techniques.

In [34] the classification of masked image data involves categorizing images that have undergone some form of masking or obscuring. This could include images where certain parts are obscured intentionally or unintentionally, such as due to privacy concerns or noise. The task typically entails developing algorithms or models capable of accurately identifying the underlying content of the masked images despite the obfuscations. Efficiently handling masked image data is crucial in various domains, including computer vision, medical imaging, and security applications. Techniques for classification often involve advanced image processing, pattern recognition, and machine learning algorithms to infer the true content of the images from the masked information.

### II.III. Enhancing Vision-and-Language integration

In [35], a method is discussed to address the challenges posed by the size of Vision-and-Language Pretraining (VLP) models in image-text retrieval tasks. Introducing Dynamic Contrastive Distillation, the authors aim to improve the performance of real-world search applications by limitations posed by the large size of VLP models.

Coca is an image-text foundation pretraining model capable of undertaking various vision and vision-language tasks [36]. This model’s architecture includes an image encoder, a text encoder, and a decoder specifically designed for generating captions for images. The training process of the model focuses on two primary objectives: a contrastive loss, which aligns the image and text embeddings within a unified space, and a captioning loss, which predicts text tokens based on the provided image and preceding tokens. This model gains advantages from pretraining on extensive alt-text data and annotated images. Furthermore, it’s adaptable to downstream tasks through zero-shot, few-shot, or fine-tuning methods. Empirical evidence demonstrates that Coca outperforms its competitors in several benchmark tests, including those on ImageNet, MSCOCO, VQA, and NLVR2.

In [37], the refinement of the pre-existing architectural framework HybridBranchNet is addressed. The core of the improvements lies in adapting the architecture based on empirical results derived under a range of testing conditions. A key challenge identified is the dependency on single-label feature extraction in the processing of images that contain multiple object categories. This method falls short as it often results in errors, particularly when the architecture encounters images that include overlapping or similarly structured classes. To overcome these limitations, the proposed approach involves a more nuanced management of the extracted features. This includes incorporating additional labels or supplementary information for each image within the architecture. These enhancements aim to significantly increase the accuracy of classification, especially in instances where the extracted features present conflicting information. Further, the article emphasizes the importance of refining architectural inputs, echoing insights from preceding research in the field. It notes that different architectural designs come with different requirements. For example, some architectures might need to process images at different resolutions, while others may benefit ancillary data, like edge detection or external knowledge sources, to improve algorithmic efficiency.

A notable objective of this research is to employ simple, non-deceptive techniques to enhance classification accuracy. This is achieved by classifying images based on an interpretation of the scene and examining the effectiveness of this method across diverse scenarios. Our findings indicate that the application of appropriate reinforcement learning techniques can significantly boost classification accuracy. In the architectural frameworks we have examined, the limitations can be broadly summarized into four key areas, each presenting unique challenges to the advancement and application of artificial intelligence and deep learning technologies:

**Data Availability and Quality:** The challenges related to acquiring large volumes of high-quality, labeled data, and the implications for model training and accuracy.
**Model Robustness and Generalization:** Vulnerabilities including overfitting, susceptibility to adversarial attacks, and difficulties in generalizing from training to novel or complex real-world scenarios.
**Architectural and Integration Challenges:** Limitations in handling multi-object scenes, integrating diverse data types (e.g., visual and textual information), and achieving efficient computational performance.
**Ethical and Operational Concerns:** The need for ethical considerations in AI deployment and the requirement for models to be adaptable and reliable across various applications and conditions.

The overarching goal is to facilitate the transfer of knowledge from images directly into the model, thereby enhancing the model’s performance in scenarios that differ from the initial training data conditions. If this knowledge transfer proves effective, it could open the door to designing more sophisticated architectural models that require less data input yet maintain high accuracy. Such models would be versatile, and capable of delivering reliable performance across various conditions and hardware configurations, marking a significant advancement in the field of image classification and machine learning. As a summary, a list of different methods for image classification is provided in Table 1, along with their advantages and disadvantages, and the dataset used for each method.

**Table 1:** Methods of image classification with advantages, disadvantages, and utilized datasets.

Method	Advantages	Disadvantages	Dataset
Deep Learning is Robust to Massive Label Noise [22]	Robust to incorrect labels, can improve generalization	Requires large datasets, may be computationally intensive	Large datasets with significant label noise (MNIST, CIFAR, ImageNet)
Multiview semi-supervised learning model for image classification [23]	Utilizes unlabeled data, can leverage multiple data views	May struggle with highly disparate views, semi-supervised learning can be sensitive to noise	Multiview datasets with structured graph information (HeriGraph)
A comprehensive system for image scene classification [24]	Can handle complex scenes with multiple elements	May require significant computational resources, complex to implement	Image datasets with varied scene content (VAST)
Cold-start active learning for image classification [25]	Efficient for small datasets, can quickly adapt	Initial model performance may be low, and requires careful selection of samples	Large unlabeled datasets with class imbalance (SODA10M, ImageNet-21K, JFT-300M)
A smart waste classification model using hybrid CNN-LSTM with transfer learning for a sustainable environment [26]	Effective for temporal and spatial data, benefits from transfer learning	May need fine-tuning for specific waste types, complex model architecture	Datasets of waste images with labeled categories (TrashNet)
Hybrid CNN-LSTM model with efficient hyperparameter tuning for prediction of Parkinson’s disease [27]	Efficient hyperparameter tuning, good for biomedical signals	Specific to Parkinson’s disease, may not generalize well to other conditions	Datasets of Parkinson’s disease patient data with clinical attributes
A similarity-based framework for classification tasks [28]	Can be very accurate, and useful for fine-grained classification	Depends on the quality of the similarity measure, may not scale well	Multi-label datasets with diverse classes
CoAtNet: Marrying Convolution and Attention for All Data Sizes [29]	Versatile for different data sizes, combines strengths of CNNs and attention	May be complex to train, and requires careful architecture design	ImageNet, ImageNet-21K, JFT-300M
A Hybrid Geometric Spatial Image Representation for scene classification [30]	Innovative approach to scene classification	May require extensive training data, and geometric features can be complex to extract	ADE20K, SUN database
Classification of masked image data [31]	Useful for incomplete or occluded images	Performance can be affected by the extent of masking, may require additional processing	COCO, VOC2012

## III. Proposed method

We aim to tackle the inability of deep neural networks to autonomously comprehend fundamental concepts and semantic relationships between objects. Despite their high accuracy in object classification, current networks often fail to grasp the complex concepts underlying images. Our approach, "HybridBranchNetV2", integrates linguistic knowledge with image processing techniques. Initially, we assess both image and text processing capabilities. Image processing identifies and extracts visual features, while text processing understands the associated concepts and terms. By fusing these domains, HybridBranchNetV2 achieves a deeper understanding of image content, identifying visual features and comprehending the semantic relationships within images. This results in a model capable of classifying objects with greater accuracy.

The HybridBranchNetV2 addresses key challenges in image classification, including the lack of inherent content knowledge within images, the necessity for large datasets, and the complexities in interpreting deep neural networks’ operations. By combining linguistic knowledge with advanced image processing techniques, this method enhances classification accuracy without needing to adjust image resolution or increasing the number of parameters, making it more efficient. Our approach significantly improves image classification accuracy by integrating textual and visual information, ensuring a comprehensive understanding of image content. Unlike deep neural networks relying solely on single labels, hybridbranchnetv2 transfers essential knowledge to the system, overcoming the limitations of single-label-dependent networks and improving classification accuracy. Additionally, we incorporate reinforcement learning to optimize pattern extraction, leveraging its unique capabilities to enhance the system’s ability to identify and interpret patterns within images. This integration adaptively enhances feature extraction, playing a critical role in fine-tuning the overall classification process.

**Fig 1:**
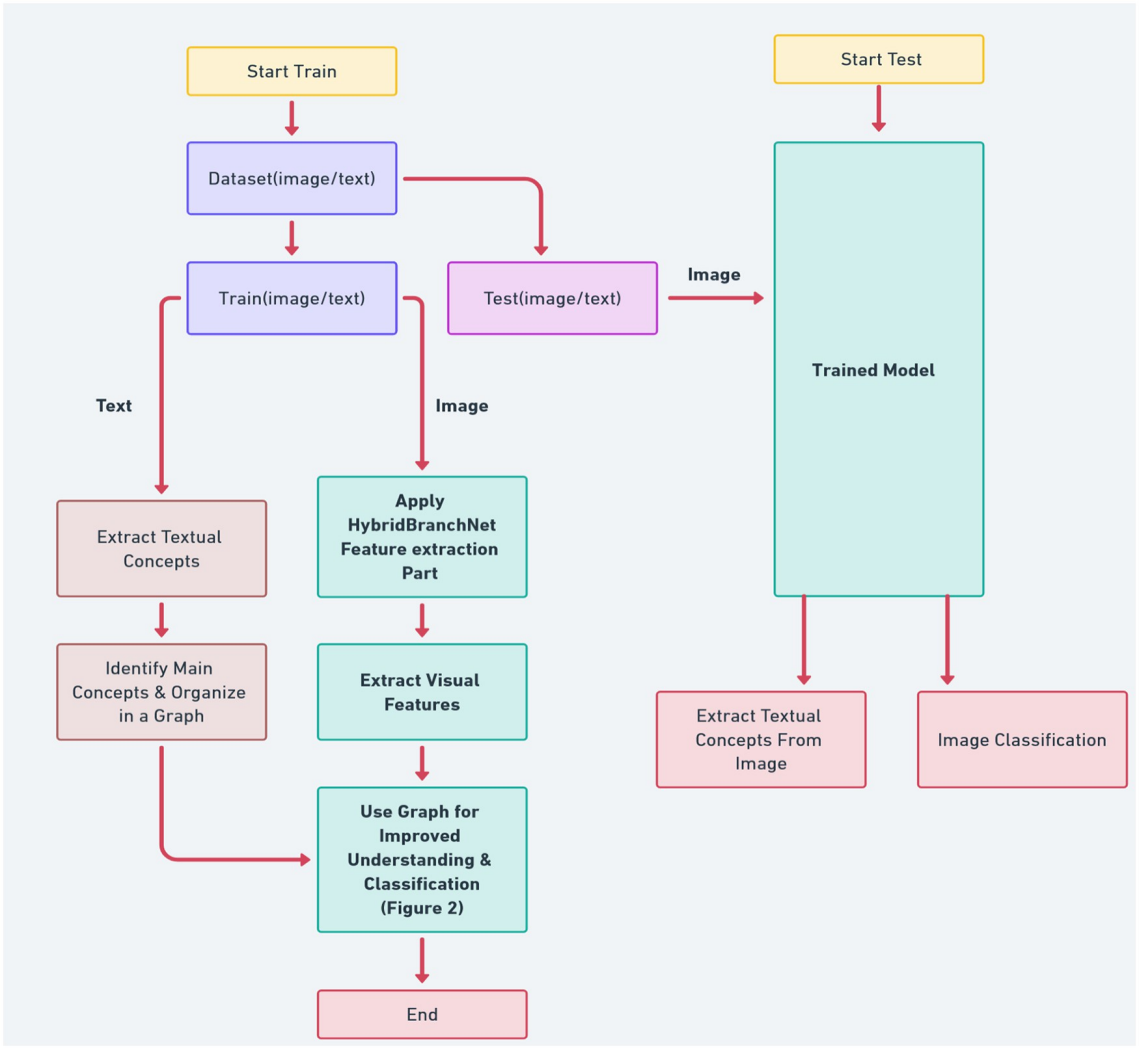
Image classification process using HybridBranchNetV2 model and graphical concept analysis.

**Fig 2:**
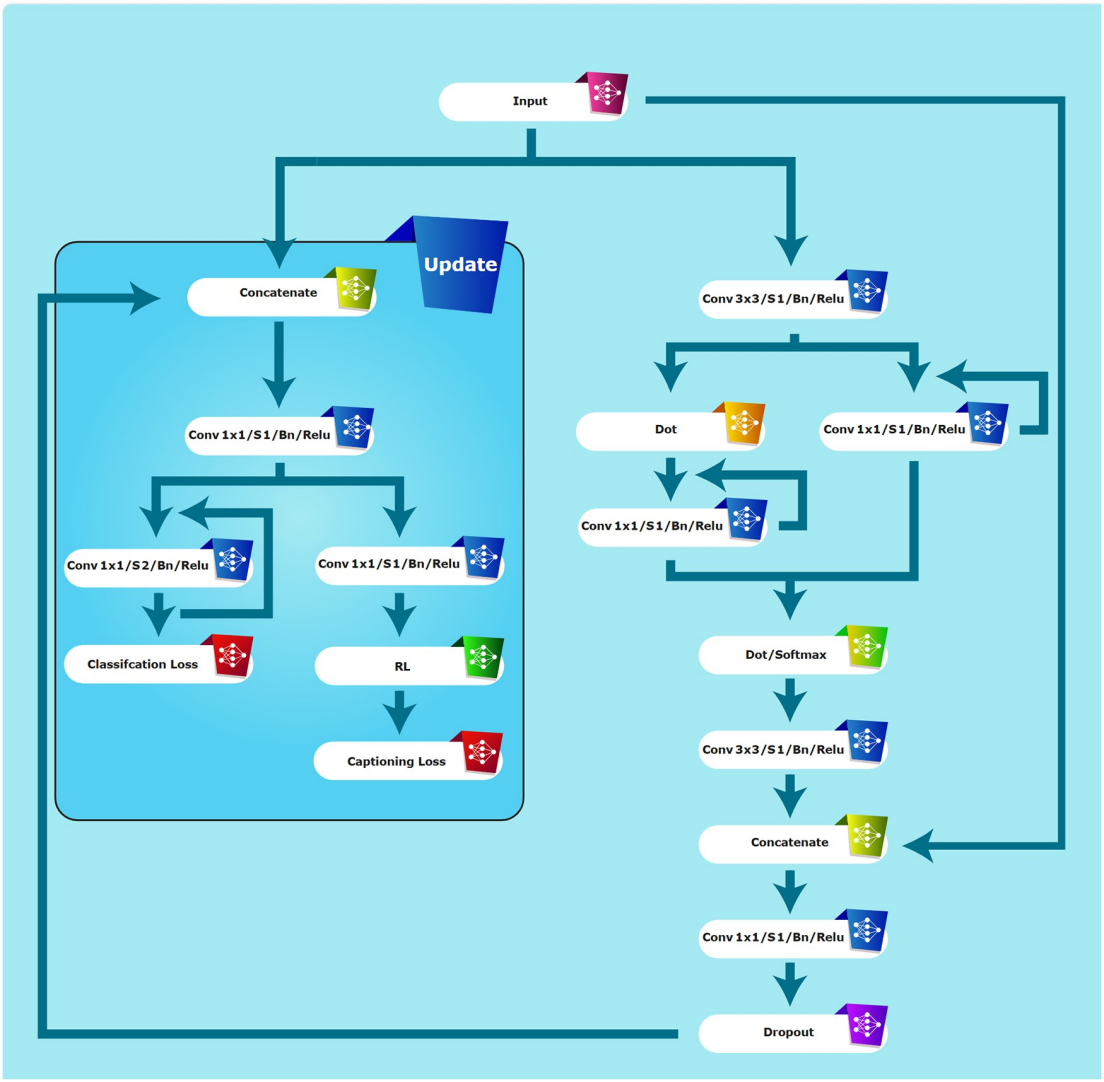
Architecture for updating hybrid feature extraction in HybridBranchNetV2.

In Fig 1, an overview of the proposed method in the paper is illustrated. The modifications, illustrated in Fig 2, play a key role in enabling the extraction of textual features from images. Additionally, the reinforcement learning component enhances both the efficiency and accuracy of the pattern extraction process. Further details on these enhancements and their implementation will be discussed in subsequent sections of this paper.

### III.I. HybridBranchNetV2 architecture

As shown in Fig 2, HybridBranchNetV2 has made modifications to the original architecture [9]. These modifications include the addition of a reinforcement learning section and the creation of a loss function based on extracting relationships between objects in the image (these are labeled as ‘update’ in Fig 2). These changes aim to create a more effective feature in the architecture leading to improved. Although considerable efforts have been made to understand the architecture’s errors, our goal has been to conduct research for the development of a stronger architecture. These efforts encompass not only crafting the methodology to construct relationship graphs for the architecture but also selecting a fitting loss function for these graphs.

To strengthen the emphasis on relationship-centric aspect of our methodology, we introduced an advanced loss function designed to capture nuanced relationships between objects within the image. Specifically, we implemented the Triplet Margin Loss, a function designed to enhance the network’s ability to discern relationships, thereby contributing to the overall robustness of our architecture. The Triplet Margin Loss is denoted with Eq (1).


L(y_anchor,y_positive,y_negative)=max(0,‖yanchor-ypositive‖2-‖y_anchor-y_negative‖^2+α) (1)

Let us define the key terms: "*y_anchor*" is the embedding vector for the anchor sample, "*y_positive*" is the embedding vector for the positive sample, "*y_negative*" is the embedding vector for the negative sample, "*α*" is the margin, a constant value. The Triplet Margin Loss function, denoted as L, is designed to optimize the distances within the feature space for image classification. The vector representation (y) encompasses information related to the features of the image, which can include shapes, colors, textures, and relationships between objects within the image. In the context of the HybridBranchNetV2 architecture, the feature space is particularly designed to comprehend and extract relationships between objects in the image. However, this space is not only related to the objects and their visual features but also to the input text, as the text can contain descriptions or labels that aid in a more accurate interpretation of the image.

This elaboration on the feature space, its relation to the input image and text, underscores the importance of a well-defined feature space in enhancing the model’s ability to discern intricate relationships within the data, thereby bolstering the architecture’s robustness and effectiveness in image classification tasks. It achieves this by minimizing the squared distance between the anchor and positive samples, while simultaneously maximizing the squared distance between the anchor and negative samples. This is done with a margin of at least "*α*" to ensure distinct separation. The function employs the term *max* (0, ∥*yanchor − ypositive*∥2−∥*yanchor − ynegative*∥2+*α*) to ensure that the loss remains non-negative. It activates only when the squared distance between the anchor and positive points minus the squared distance between the anchor and negative points, plus a margin α, fails to meet the set criteria.

The proposed function shares conceptual similarities with reinforcement learning algorithms, particularly in how it enhances performance. Its effectiveness lies in creating a competitive dynamic between negative and positive outcomes, aimed at widening the gap between features linked to these instances. Through extensive experimentation with various loss functions, it was observed that our proposed method, incorporating this Triplet Margin Loss, consistently yielded the highest accuracy. This approach effectively leverages the principles of reinforcement learning, translating them into a robust framework for image classification.

The development of a graph for image description is crucial because it establishes the core framework for the whole process of analyzing and describing images. This graph-based structure is key for defining how objects, entities, and their relationships are represented and connected in an image. It serves as the backbone for a thorough understanding and interpretation of the scene, ensuring a coherent and detailed analysis of the visual content. Incorrect execution of this process can lead to serious consequences. The graph’s accuracy directly affects how well the neural network performs in analyzing images. This network relies on the encoded information from the graph to interpret the image and produce relevant descriptions or predictions. A graph that’s not accurate or has errors can lead to misinterpretations, misclassifications, and reduced performance [38]. Additionally, a faulty graph can obstruct network convergence during training. Training a neural network involves adjusting its internal parameters to minimize the difference between predicted and actual results. so, if the graph’s representation has inconsistencies, achieving convergence can become challenging. This challenge can lead the model to longer training times, reduced accuracy, or even training failure. It is important to highlight the significance of creating an accurate graph because it strongly affects the effectiveness of image analysis systems. Its role is crucial in ensuring the system’s reliability and precision, marking it as an indispensable element.

The graph, in our model, refers to a structured representation that captures the relationships and interactions between various objects and entities within an image. This representation is not a mere collection of nodes and edges; rather, it is a sophisticated framework designed to encode the spatial and semantic relationships among the depicted elements. Each node in the graph represents an object or entity identified within the image, while the edges denote the relationships between these nodes, such as adjacency, containment, or any other relevant spatial or semantic interaction.

In the HybridBranchNetV2 architecture, the graph is incorporated at a critical juncture where it serves as a foundational element for analyzing and understanding the image content. The process begins with the extraction of features from the input image, which are then used to construct the graph. This involves identifying objects within the image, determining their attributes, and establishing the relationships between them based on their spatial arrangement and semantic connections. The constructed graph thus provides a comprehensive and interconnected representation of the image, enabling the architecture to analyze the image in a more structured and relational manner.

The significance of the graph in our model lies in its ability to facilitate a more nuanced and detailed analysis of the image. By leveraging the graph-based structure, our architecture can effectively interpret the scene depicted in the image, taking into account not only the individual objects but also the complex web of relationships that connect them. This leads to a more accurate and thorough understanding of the image content, enhancing the model’s capability to generate relevant descriptions or predictions.

Moreover, the accuracy of the graph directly impacts the performance of the neural network in analyzing images. An accurately constructed graph ensures that the network has access to a rich and correctly encoded dataset of relationships and attributes, which is crucial for interpreting the image effectively. Any inaccuracies or errors in the graph can result in misinterpretations, misclassifications, and ultimately, reduced performance of the model.

In summary, the graph is a central component of our model, integrated into the HybridBranchNetV2 architecture as a means to encode and analyze the relationships and interactions between objects within an image. Its role is instrumental in achieving a deeper and more precise understanding of the image content, thereby enhancing the overall effectiveness and accuracy of the image analysis system.

### III.II. Key features of the Visual Genome dataset

The "Visual Genome" dataset stands out as one of the most distinguished and exhaustive datasets in computer vision and image interpretation domains. Crafted by the deep learning and image understanding research community, this dataset provides detailed and accurate information for a variety of applications involving different images [15]. Here, we summarize key features of this dataset, but full descriptive details are provided in Section IV.I.

- **Image Descriptions:** This dataset encompasses elaborate descriptions for various images. These descriptions articulate the specifics of the image content and characteristics of objects, attributes, relationships, and different regions within the image.
- **Object Attributes:** Visual Genome offers extensive insights into the attributes of objects present in the images. These attributes encompass features such as color, shape, size, and material of objects.
- **Object Relationships:** The dataset contains information regarding relationships between objects in the images. These relationships include spatial relationships like on, beside, inside, and above.
- **Description of Image Regions:** Visual Genome provides detailed descriptions of various regions within the images, described using natural language sentences.
- **Question and Answer Pairs:** This dataset comprises a collection of questions and corresponding answers related to the images. These questions can aid in the interpretation and description of the images.

### III.III. Graph-based image analysis with selective focus and relationship mapping

To capture the complexity of images in a structured manner, our approach utilizes a graph-based framework. This methodology constructs a network where each identified object is represented as a node, allowing dynamic interaction by adjusting the activation state of these nodes. This mechanism allows for a detailed examination of the image starting from identifying objects to understanding their characteristics and relationships. The adoption of this model is motivated by the need for a robust and flexible language model that can work within strict time limitations. By engaging with four key lines of inquiry—naming objects, assessing their dimensions, enumerating their features, and pinpointing their spatial interconnections—we enable the model to delve into a wide range of scenarios, thereby enhancing its interpretative capacity. Consequently, this strategic approach not only enhances the diversity of the system’s analytical capabilities but also ensures a comprehensive extraction and description of the features defining each object in the image.

To improve our image analysis strategy, we design a system where each object, represented as a geometric shape like a circle, is encoded with a unique identifier, such as "00001". This designation facilitates the establishment of binary connections between objects. To standardize the input, the coordinates of objects’ bounding boxes are normalized to a range between zero and one. Also, normalizing and assigning unique numbers to shapes, establish a consistent descriptive framework using numerical identifiers. This framework ensures uniform object representation across different images.

Moreover, we enhance our graph structure to enable selective focus within images. By leveraging the bounding box coordinates, we can isolate specific segments of an image while maintaining the relationships between objects within this cropped view. Also, we might choose to concentrate on a subset of objects. In doing this, additional connections are excluded and recalibrating the graph to focus exclusively on a subset of objects. This selective emphasis not only increases attention to specific object relationships but also expands the range of image augmentation possibilities. As a result, the network can be trained on a diverse set of scenarios, enhancing its ability to generalize.

The method described above relies on using numerical identifiers to accurately and represent shapes and their relationships in images. This approach is crucial because it transforms visual information into a quantifiable format, making easier for our network to process. It is important to note that while the original graph captures all relationships within an image, our modification allows for the selective removal of specific connections. This allows us to customize the graph for more focused analysis. Such selective pruning is not a contradiction but a strategic choice. It helps to highlight pertinent relationships while training the network across various scenarios, thereby improving its capacity to generalize from specific instances to broader contexts.

Once the proposed architecture has been finalized, the input data prepared, and the input graph initialized, we proceed to the training phase of the proposed network. This process will be outlined in three distinct stages, each of which will be detailed in the following sections.

The Visual Genome dataset encompasses various sources of noise and complexity in the domain of computer vision, some of which include:

- Diversity in lighting conditions and viewpoints: Images are captured from various sources and may exhibit different lighting conditions and viewing angles, potentially impacting the model’s accuracy. However, this diversity also fosters appropriate generalization since the images are captured under various conditions.
- Diversity of objects and their positions: The presence of numerous objects and their varying positions in images can introduce further complexity in image interpretation and model accuracy. With over 34,000 available classes in the dataset, using a model with fewer parameters may hinder the extraction of strong features. Therefore, employing a reinforcement learning model could assist in the suitable features extraction, managing this issue to some extent by emphasizing specific feature extraction during the reinforcement learning phase.
- Diverse descriptions and precise details: Some descriptions may focus more on specific parts of the image or provide more detailed information, which can differentiate the model. However, some of these descriptions may also contain noise, such as additional information or errors, which are present in the utilized dataset.
- Discrepancies in object appearances and features: Objects may differ in shape, size, color, and other features, potentially leading to challenges in modeling them effectively.
- Inaccuracy in labeling: As indicated in the data descriptions, a large number of objects, features, and relationships in images are labeled, which leads to inaccuracies in labeling.
- Rare and unintended occurrences: In large datasets like Visual Genome, rare, unintended occurrences, or data that do not belong to primary categories may exist, contributing to model challenges.
- Imbalanced data: Some images may have fewer or more objects or relationships between objects compared to others, potentially causing data imbalance and transferring this imbalance to the model.

Considering these challenges and noises, the importance of thorough preprocessing and corrective measures on the data, as well as training the model with high accuracy, becomes more apparent. The preprocessing steps we conducted on the dataset included:

- Removing classes and features that were less utilized, normalizing the dataset to around 21,000 classes that were utilized more frequently, and discarding the remaining classes.
- Employing augmentations such as rotation, channel swapping, cropping, brightness adjustments, and adding optical noise to the images.
- Utilizing reinforcement learning to enhance feature extraction during training.

### III. IV. The proposed network training

The proposed neural network training consists of 3 stages, which are described below.

**Stage 1:** In the initial phase of our work, we utilized the HybridBranchNet architecture, version 3 as cited in [9], to classify objects. This architecture notable for its fewer parameters and the incorporation of a branching technique along with hyper-feature aggregation. This combination results in improved accuracy in object classification compared to similar models. We improved this architecture by expanding upon the features derived from the hyper-branch. It interfaces with multiple layers of neural networks and is linked to a specific loss function. This arrangement allows for refining features within the network, equipping it with the proficiency to interpret and respond to queries related to the objects in the images.
**Stage 2:** To enhance the precision of our network, we have integrated a reinforcement learning algorithm, which aids in selecting the most accurate responses from the relationship matrix established in the previous step. This integration is crucial as it allows the network to iteratively improve its accuracy in deducing responses. Prior to applying reinforcement learning, the network struggled with extended periods of difficulty in achieving convergence.

By accurate evaluation of the relationship matrices within the graph, we found that a fine-tuned optimization approach, based on a reward-and-penalty system, was necessary. This approach supports a consistent and progressive rate of convergence by reinforcing correct predictions and discouraging erroneous ones. it guides the network towards more accurate outcomes over time. The substantial difference in results obtained without the use of reinforcement learning is highlighted in Table 6 of the results section, illustrating the significant role this method plays in the network’s performance.

The method ensures a consistent and steady convergence rate by providing a structured framework for the network to learn from its actions. As the network makes predictions, the reinforcement learning algorithm evaluates these predictions and either rewards or penalizes the network accordingly. This feedback loop establishes a systematic path for the network, minimizing oscillations and errors that typically occur in the learning process. It results in a more stable and reliable convergence towards the correct solutions.

**Stage 3:** The remaining segments of the HybridBranchNet architecture, dedicated to classification, are employed to detect object categories, enhancing the precision of object category identification within images.

### III.V. Reinforcement learning optimization using Q-learning algorithm

The Q-learning algorithm has been selected due to its stability and off-policy capabilities. This method allows for the gradual collection of more information from the environment, enabling the formulation of optimal decisions without the need for complex policies. Its simple structure and ease of implementation, combined with the use of neural networks for estimating Q-values in complex environments, make this algorithm suitable for addressing the problem at hand. Additionally, Q-learning strikes a good balance between exploration and exploitation, which is crucial for gradual and stable optimization. In the proposed approach, reinforcement learning, particularly through the Q-learning algorithm, has been employed to extract complex and hidden features from images. This algorithm effectively optimizes interactive behaviors in environments where an accurate model is lacking. The objective of HybridBranchNetV2 is to extract intricate features that can aid in identifying latent relationships between objects and various parts of an image. Furthermore, a relational matrix has been designed to represent the connections between objects and different segments of the image, enhancing the understanding of interactions within the image. Reinforcement learning assists in learning which changes in this matrix can enhance classification accuracy. Through Q-learning, the model can optimize itself via positive and negative feedback, discovering the best patterns for object recognition in images. Ultimately, this method provides an interactive and adaptive process that facilitates the optimization of high-level features and their improvement based on the complex relationships between objects and features within the image. This advantage is particularly significant in tasks that require a deeper understanding of intricate relationships in images.

In the optimization of the HybridBranchNetV2’s pattern extraction process, reinforcement learning (RL) plays a critical role, especially through the employment of Q-learning, a model-free reinforcement learning algorithm. Q-learning assists the model in identifying which actions to take in a given state based on the action’s expected utility to achieve the goal, which, in this context, is the accurate classification of objects in images. During the development and optimization of the proposed model, experiments were conducted to assess the impact of different approaches on the overall system performance. Part of these experiments involved examining the accuracy of the proposed architectures, the results of which indicated no significant improvement in performance. To further improve the model, textual features were added to the model. However, analysis of the results revealed that these changes only marginally improved the accuracy of the model, as shown in Table 6.

We performed a deeper analysis on the feature extraction process. By employing heatmap techniques to examine the model activations in different layers, we were able to identify strengths and weaknesses in feature extraction. The proposed model based on reinforcement learning was able to focus on key objects and activate them. This focus and activation without reinforcement learning was limited and resulted in poor feature extraction. A stronger reason for the improved performance in image classification after employing reinforcement learning lies in its ability to provide the model with increased feedback in an interactive environment, enabling it to experience new insights. This feedback may include information obtained by the model during decision-making processes regarding the images. Additionally, through the selection of defined actions, the model can perform newer operations on features and identify better patterns in the images, ultimately improving the crucial features for classification.

These advancements are denoted in Fig 5, where heatmaps demonstrate the model’s ability to identify and focus on key objects. The heatmaps show that the critical features in the model’s decision-making process are better extracted using reinforcement learning.

Q-learning operates on the principle of Q-values or action-value pairs, which are estimates of the expected utility of taking a certain action in a given state. The Q-value for a state-action pair (s, a) is denoted as Q(s, a) and is updated with Eq (2).


Q(s,a)=Q(s,a)+α*(R(s,a)+γ*max(Q(s′,a′))-Q(s,a)) (2)

where:

- “*s*” is the current state.
- “*a*” is the action taken.
- “*s*’“ is the new state after taking action “*a*”.
- “*R*(*s*, *a*)” is the reward received after taking action “*a*” in the state “*s*”.
- “*α*” is the learning rate (0 < *α* ≤ 1).
- “*γ*” is the discount factor (0 ≤ *γ* < 1), which determines the importance of future rewards.
- max over “*a*′” of *Q*(*s*′, *a*′) represents the maximum Q-value achievable in the new state “*s*‱”, over all possible actions “*a*′”.

The agent updates Q-values by interacting with the environment repeatedly. It takes actions, observes state changes, and considers the rewards in the process. This iterative process is like exploration, where the agent aims to enhance its policy. The policy is the strategy for selecting the best action in a given state.

### III.VI. Visualization of the reinforcement learning process

To gain a deeper understanding of the impact of reinforcement learning on the decision-making process within the proposed neural network, a step-by-step explanation of how feature extraction and classification are optimized during the training process using the Q-learning algorithm is provided below:

1. Initial Feature Extraction: The process begins by defining an initial state, where features are extracted using the HybridBranchNet architecture. These features are then sent to the reinforcement learning module for evaluation and the extraction of new features aimed at enhancing the model’s performance.
2. Action Selection: The extracted features, considered the current state, are forwarded to a fully connected neural network. This network selects an appropriate action and generates a new state, representing the newly extracted features derived from the initial data.
3. Reward Evaluation: The reinforcement learning module evaluates the outcome based on the executed action (i.e., adjustments or modifications to the features). This evaluation is conducted by comparing the predicted classifications or relationships with the actual outcomes. If the classification or identification of relationships between objects is accurate, the model receives a positive reward; otherwise, a penalty is imposed.
4. State Transition: Following the feedback (reward or penalty), the model updates its state and adjusts the Q-values associated with the executed action. This enables the model to learn which modifications to the features can enhance classification accuracy.
5. Feedback Loop: This process continues iteratively, allowing the model to continually optimize the features and improve its output. In each iteration, the Q-values are updated, and a better policy for feature extraction that maximizes the model’s accuracy is learned.
6. Final Output of the Reinforcement Learning Network: The final result of each feature extraction cycle is produced by the reinforcement learning network as an updated set of features. This final output is applied to the model’s loss function to implement necessary improvements in the accuracy and quality of feature extraction throughout the training process. Consequently, the HybridBranchNet operates more effectively in conjunction with the reinforcement learning network at every stage of training.
7. Convergence: As the training process advances, the reinforcement learning module assists the model in achieving a more stable and accurate strategy for feature extraction and classification, thereby reducing fluctuations and improving overall accuracy.

**Fig 3:**
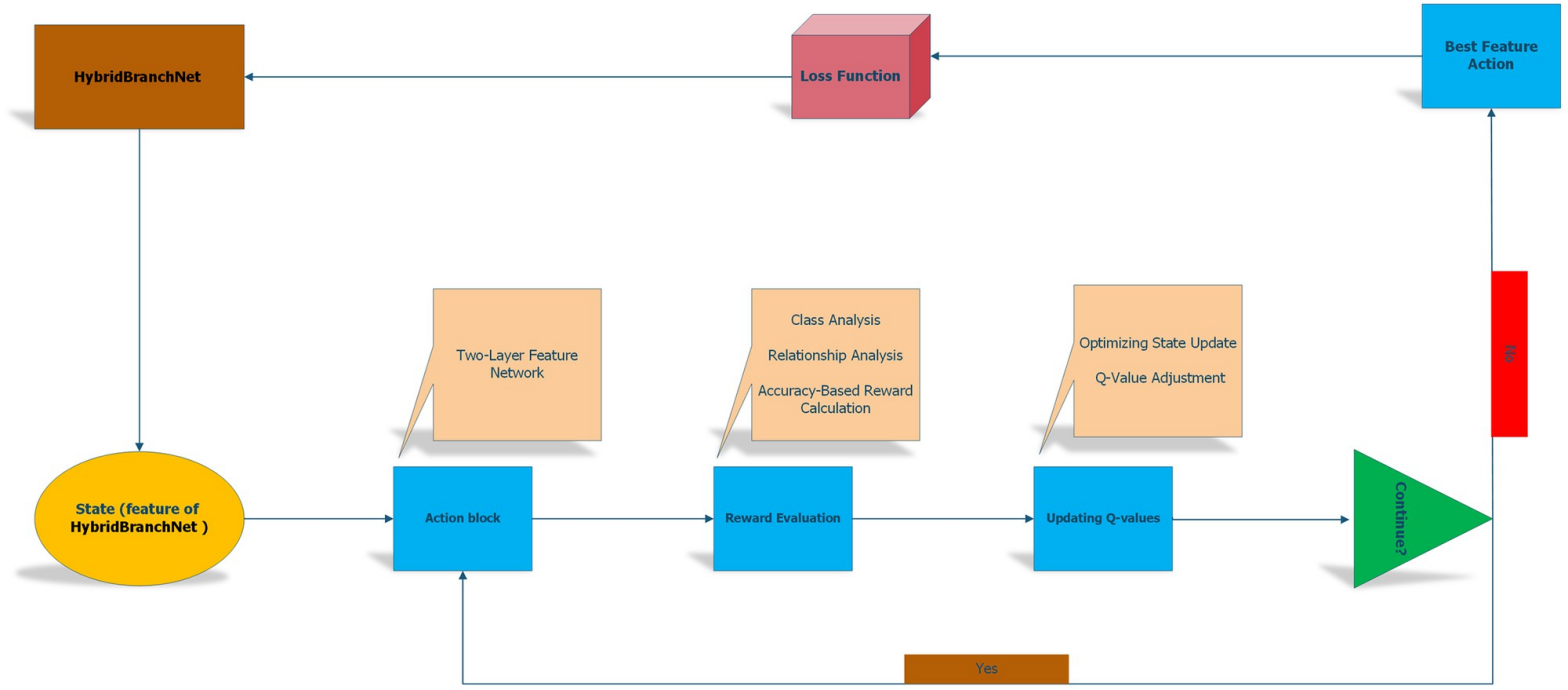
Reinforcement learning in image classification: Feature extraction and categorization process.

Fig 3 provides a concise depiction of the reinforcement learning application for image classification. It outlines the process by which the reinforcement learning model iteratively improves feature extraction to categorize images more accurately. The figure demonstrates the progression from initial feature identification to the conclusive categorization step. It highlights how the reinforcement learning framework contributes to guiding the network *t* for more accurate image classification through reward-driven optimization.

### III.VII. State and rewards

To integrate Q-learning into the HybridBranchNetV2 model, we simulate an environment where each state represents a specific pattern of features extracted from images, and actions correspond to adjustments in the feature extraction process. Rewards are assigned based on the accuracy of the object classification. Positive rewards are given for correct classifications, while negative rewards are assigned for incorrect ones. Through iterative training, the model learns a policy that maximizes the total reward, which is equivalent to improving the accuracy of image classification. The RL framework complements the existing architecture by introducing an adaptive mechanism that enhances the decision-making process of the neural network, leading to a more robust and accurate image classification system.

The integration of Q-learning into HybridBranchNetV2 enables the model to leverage not only its existing hybrid knowledge but also to dynamically refine its feature extraction and classification processes. This adaptive capability is crucial for dealing with complex and nuanced visual data, thereby pushing the limits of what’s possible with AI in the field of image classification.

In the HybridBranchNetV2 model, the ’actions’ within the Q-learning environment refer to modifications that the model can make to the feature extraction process. These modifications can encompass a variety of adjustments, such as selecting different features to focus on, altering the algorithms used for feature extraction. All these adjustments aim to enhance the accuracy of image classification.

To elaborate, when Q-learning is integrated into HybridBranchNetV2, each action taken by the model is an intentional step towards improving the pattern recognition capabilities. For instance, the model might ’decide’ to give more weight to certain visual features if doing so has previously led to successful object classification. Conversely, it might ’learn’ to disregard or downplay features that have consistently contributed to misclassification. This can involve changing adjusting thresholds for feature significance, or even selecting entirely different sets of features for subsequent processing stages.

These action choices are influenced by a reward system that prioritizes classification accuracy. Positive rewards are allocated for correct classifications, reinforcing the link between specific actions and positive outcomes. Conversely, negative rewards are assigned to incorrect classifications, prompting the model to adjust its approach. Through this iterative refinement process, the model progressively enhances its feature extraction capabilities, leading to a more refined and accurate image classification method.

Subsequently, with the establishment of this baseline performance, reinforcement learning was systematically deployed at the epoch level to facilitate a targeted optimization of the network’s feature extraction capabilities. The reinforcement learning algorithm systematically assessed and improved the significance of specific features through a process of weight adjustment. This strategic modulation aimed to enhance the discriminative power of the network, thereby improving the precision of object classification tasks.

This integration of reinforcement learning into the network’s training regimen catalyzed an iterative refinement process. Each iteration was carefully designed to refine and reinforce the network’s ability to recognize and prioritize informative features, gradually improving classification accuracy. The cumulative enhancements achieved through this adaptive learning process were captured and quantitatively analyzed, with the detailed findings and their implications for the model’s performance encapsulated in Table 6. This table provides empirical evidence of the effectiveness of reinforcement learning in enhancing the deep neural network’s classification skills after the initial non-reinforcement learning phase of training.

The strategic introduction of reinforcement learning thus served to transition the network from a state of static learning to a dynamic learning curve, underlining the synergy between pre-established network knowledge and the adaptive insights garnered through reinforcement learning.

## IV. Experimental results

In this section, we compare the results of the proposed architecture with various object classification methods. The primary objective of these experiments is to demonstrate the capability and efficiency of the proposed method in the field of object classification when applied to unfamiliar data. We believe that the system’s classification accuracy will be higher when it performs better under unfamiliar conditions. Furthermore, our results are based on training and testing data from a specific dataset. It’s important to note that the main goal of this architecture is not to extract text from within the image; instead, it leverages unique features associated with object relationships to extract more potent features.

The experiments were carried out on a system equipped with an Intel Core i9-10900K Processor, which has a base clock speed of 3.70 GHz and can boost up to 5.30 GHz using Intel Turbo Boost Technology. It includes 20 MB of Intel Smart Cache. The graphical processing was handled by an NVIDIA Tesla V100, known for its robust computing capabilities, especially in deep learning and scientific computing, featuring 32 GB of HBM2 VRAM. The system was supported by 64 GB of DDR4 memory at 3200 MHz and had storage capabilities provided by a 2 TB NVMe Solid State Drive, with additional data storage facilitated by a 4 TB Hard Disk Drive.

On the software front, the system ran Ubuntu 20.04 LTS as its operating system. The programming was primarily conducted in Python 3.8, making extensive use of the PyTorch 1.8.1 deep learning framework, which was chosen for its CUDA 11.1 support to enable GPU acceleration. Additional libraries such as NumPy were used for numerical operations, Matplotlib for graphical plotting, and OpenCV for handling image processing tasks. The development and testing of the model were facilitated through the use of Jupyter Notebook, which provided an interactive development environment.

This experimental setup, leveraging the NVIDIA Tesla V100 GPU for its exceptional computational power, was meticulously selected to satisfy the high computational demands of training and evaluating the HybridBranchNetV2 architecture. The initial training phase, conducted over approximately 30 epochs without the integration of reinforcement learning techniques, established a solid foundation for the network to achieve its maximum performance under the given parameters. This base level of performance was critical for the subsequent application of reinforcement learning, ensuring that the network was adequately prepared for this advanced learning approach. The detailed specification of the hardware and software used in these experiments is intended to provide clarity and facilitate the reproducibility of our results, adhering to the scientific standards for experimental documentation.

The design of our proposed neural network model, HybridBranchNetV2, is available at the following GitHub links: https://github.com/Eparcham/HybridBranchNetV2. This repository contains all the necessary information, including the model architecture.

### IV.I. Introduction to datasets

In this section, we provide an overview of the datasets utilized in this study.

1. **Visual Genome** dataset consists of over 108,000 images, with each image containing an average of 35 objects, 26 attributes, and 21 pairwise object relationships. This dataset also includes region descriptions and questions with corresponding answers. According to a scientific paper [15], the number of different classes in the Visual Genome dataset is as follows:
  - Number of object classes: 33,877.
  - Number of attribute classes: 18,291.
  - Number of relationship classes: 6,672.
2. **CIFAR-100** is a dataset used for image classification tasks. It contains 100 classes, each with 600 images. It is often used for evaluating the performance of deep learning models in fine-grained image classification.
3. **Flowers-102** is a dataset specifically designed for fine-grained image classification. It consists of 102 different categories of flowers, with a total of over 8,000 images. This dataset challenges models to distinguish between visually similar flower species.
4. **ImageNet 1K**, often referred to simply as ImageNet, is one of the most widely used datasets in computer vision. It contains over a million images across 1,000 different object categories. ImageNet is frequently employed for large-scale image classification and pretraining deep neural networks for various vision tasks.

In summary, these four datasets were selected to comprehensively evaluate the versatility and performance of our algorithm. Each dataset poses its unique challenges: Visual Genome with its complex relationships, CIFAR-100 and Flowers-102 with classification intricacies, and ImageNet with its vast number of categories. This range of challenges ensures a comprehensive assessment of our algorithm’s capabilities.

### IV.II. Implementation results

In this section, we explore the detailed outcomes and noteworthy findings from our thorough experimental implementation and comprehensive evaluation of the previously introduced datasets. Tables 2 and 3 showcase the accuracy results of our proposed method, HybridBranchNetV2, compared with other well-established models on the CIFAR-100 and Flowers-102 datasets. This comparison provides a clear insight into our model’s performance against state-of-the-art methods. On the CIFAR-100 dataset, HybridBranchNet3 achieved the accuracy of 92.30%. Meanwhile, HybridBranchNetV2 surpassed all other models, registering an accuracy of 98.25%. Notably, despite its fewer parameters (approximately 16M), HybridBranchNetV2 demonstrated superior accuracy, highlighting the efficiency of our architecture in image classification tasks, even when compared with larger and more complex models like EfficientNet-L2 and MViT-B-16.

**Table 2:** Accuracy in CIFAR-100 dataset and comparison with other methods.

Model	Accuracy	Parameters
EffNet-L2 -A2 [39]	96.08%	≈ 480M
EffNet-L2 [39]	93.95%	≈ 37M
MViT-B-16 [40]	87.80%	≈ 67M
Oct-ResNet-152 (SE) [41]	91.70%	≈ 64M
EfficientNet-b7 Tan and Le [42]	92.26%	≈ 120M
EfficientNetV2-L [43]	92.27%	≈ 54M
EfficientNetV2-M [44]	83.64%	≈ 25M
ResNet-50 (Fast AA) [45]	83.95%	≈ 105M
HybridBranchNet3 [9]	92.30%	≈ 9M
HybridBranchNetV2	**98.25%**	≈ 16M

**Table 3:** Accuracy in Flowers-102 dataset and comparison with other methods.

Model	Accuracy
EffNet-L2 [39]	99.65%
MViT-B-16 [40]	98.50%
Oct-ResNet-152 (SE) [41]	98.21%
EfficientNet-b7 Tan and Le [42]	98.80%
EfficientNetV2-L [43]	98.80%
EfficientNetV2-M [44]	98.50%
ResNet-50 (Fast AA) [45]	97.90%
HybridBranchNet3 [9]	98.80%
HybridBranchNetV2	**99.88**

Considering that the Visual Genome dataset does not encompass all the classes found in the ImageNet dataset, we initially utilized the Visual Genome dataset to train our architecture. After this initial training, we deactivated the loss function responsible for extracting object relationships and feature-related data. This step was taken to freeze the weights associated with the relationship matrix extraction layers, ensuring no further updates to the network’s weights for these specific layers. Our objective was to equip the network with the capability to identify new classes during its training on ImageNet images. The outcomes of this training process are elaborated upon in Table 4.

**Table 4:** Accuracy in ImageNet 1K dataset and comparison with another methods train with (Visual Genome, ImageNet).

Model	Accuracy
EffNet-L2 [39]	88.3%
MViT-B-16 [40]	86.4%
Oct-ResNet-152 (SE) [41]	82.9%
EfficientNet-b7 Tan and Le [42]	84.3%
EfficientNetV2-L [43]	87.3%
EfficientNetV2-M [44]	86.1%
ResNet-50 (Fast AA) [45]	79.8%
HybridBranchNet3 [9]	83.1%
HybridBranchNetV2	**89.4%**

Based on the insights gained from Table 4 and following the initial training of the network on the Visual Genome dataset, which features a broad yet distinct class coverage compared to ImageNet, the model became adept at recognizing various object classes and their interrelationships. Later, by disabling the loss function for object relationships and features, the network shifted its focus to adapt to the new classes present in ImageNet. The outcomes reveal that our methodology allowed the network to generalize effectively, performing well at identifying these additional classes. Essentially, the results highlight that our approach, notably HybridBranchNetV2, is well-suited for handling an extensive range of object classes. This makes it an optimal choice for image classification tasks requiring expansive class coverage, competitive accuracy, and resource efficiency. Additionally, we refined our proposed architecture independently of the ImageNet dataset and contrasted the outcomes with those obtained using ImageNet. This comparison emphasized a more efficient feature extraction process. The comprehensive results of this fine-tuning are detailed in Table 5. In our results presentation, we adopted a specific notation to highlight the performance rankings of various models. The model achieving the highest accuracy is indicated using **bold font**, while the model securing the second place is denoted with underline to visually distinguish these performances at a glance.

**Table 5:** Accuracy in ImageNet 1K train model with Visual Genome.

**Model**	**Accuracy**
HybridBranchNet3 [9]	65.2%
HybridBranchNetV2	**79.5%**

To elaborate, the process involved dividing the number of images correctly identified by the algorithm (correct predictions) by the total number of images in the test dataset (total predictions). This quotient was then multiplied by 100 to express the accuracy as a percentage. This method provides a straightforward and intuitive measure of the algorithm’s overall performance.

The results presented in Table 5 provide intriguing insights when fine-tuning the proposed architecture exclusively with the Visual Genome dataset, eliminating the dependency on the ImageNet dataset.

#### Effectiveness of feature extraction

HybridBranchNetV2 notably achieved an accuracy of 79.5% when exclusively fine-tuned using the Visual Genome dataset. This outcome implies that our architecture can adeptly extract features, enabling generalization across a more expansive range of object categories without necessitating the ImageNet dataset.

For smaller datasets like CIFAR-100 and Flowers-102, we freeze the feature extraction part of the model and only train the classification head. This approach is based on the premise that the features learned from the Visual Genome dataset are highly rich and robust, allowing the model to effectively capture low-level features such as textures and colors in these smaller datasets without needing to re-train the feature extractor. The main purpose of utilizing Visual Genome is to leverage its complex relational structure to enhance feature extraction. By training on the relationships within Visual Genome, the model’s feature extractor becomes powerful enough to generalize well to simpler datasets, where the relationships between objects are not as complex.

On the other hand, for larger datasets like ImageNet, as shown in Tables 4 and 5, we fully train both the feature extraction and classification components. The results clearly demonstrate that training with both ImageNet and Visual Genome improves classification accuracy. Specifically, when the model is trained using ImageNet data that includes sufficient textual information, the classification performance is further enhanced, highlighting the complementary nature of these two datasets.

In summary, by freezing the feature extraction for smaller datasets and fully training the model for larger datasets, we are able to optimize performance across a range of classification tasks. The rich feature representation learned from Visual Genome enables effective feature extraction for smaller datasets, while full training on ImageNet allows for even higher accuracy in scenarios where both visual and textual data are abundant.

#### Model adaptability

In this setting, HybridBranchNetV2’s ability to register a markedly high accuracy, especially when compared to HybridBranchNet3’s 65.2%, underscores its adaptability and robust feature extraction prowess.

#### Reduced dependence on ImageNet

These results further accentuate that our designed architecture can reduce its dependency on the ImageNet dataset while still maintaining effectiveness. This makes it suitable for situations where procuring ImageNet data might pose challenges or be restricted.

In conclusion, the findings highlight the versatility and feature extraction proficiency of our architecture, especially evident in HybridBranchNetV2, even without depending on the ImageNet dataset. This suggests its potential for optimal performance in diverse real-world contexts where extensive labeled datasets like ImageNet might be scarce.

### IV.III. Evaluation of model performance on the Visual Genome dataset

Since there are no reports in the mentioned tables regarding the execution of the Visual Genome dataset on specific models like EffNet-L2, MViT-B-16, etc., we conducted experiments to provide a detailed comparison of the models’ ability to recognize scene features and relationships. The results of these experiments can be found in Table 6.

**Table 6:** Accuracy in Visual Genome dataset to recognize scene features and relationships.

Model	mR@20	Parameters
IETrans (MOTIFS-ResNeXt-101-FPN backbone) [13]	**36.0**	105.4
DLFE (VCTree-ResNeXt-101-FPN backbone) [46]	29.1	135.1
PCPL (MOTIFS-ResNeXt-101-FPN backbone) [47]	25.6	133.2
PCPL (VCTree-ResNeXt-101-FPN backbone) [47]	25.1	140.1
HybridBranchNetV2 [9]	29.3	**16.0**

It is important to note that our model is primarily designed for object classification, with its main emphasis not being on the extraction of precise object relationships. To evaluate the performance of our proposed model in terms of relationship extraction, we compared it with the methods presented in Table 6. These methods represent some of the state-of-the-art models proposed in recent years. As evident from the results, our method may show lower accuracy compared to some models but surpasses others. The goal of this comparison was to gauge the efficacy of our relationship extraction module and our loss function. The findings suggest that our architecture excels in extracting more potent features and knowledge.

The "mR@20" metric measures the average recall of objects in the top 20 positions of a ranked list produced by a model or system. Essentially, it measures how accurately the important objects in the scene were detected and ranked within the top 20 positions. A higher "mR@20" value indicates better performance, as it signifies that more important objects have been successfully identified and ranked higher. Mathematically, the "mR@20" metric can be expressed as in Eq (3).


$$mR@20=\left(Number\;of\;relevant\;objects\;ranked\;in\;20\right)/(Total\;number\;of\;relevant\;objects)$$ (3)

This formula, as described in Eq (3), demonstrates the ratio of correctly identified and ranked relevant objects within the top 20 positions to the total number of relevant objects in the dataset. It provides a quantitative measure of model performance in recognizing important objects within a scene. Table 7 presents a comparison of accuracy in the Visual Genome dataset with and without the application of reinforcement learning to our HybridBranchNetV2 model.

**Table 7:** Comparison of accuracy in Visual Genome dataset with and without reinforcement learning.

Model	Accuracy
HybridBranchNetV2 (Without reinforcement learning)	70.1%
HybridBranchNetV2 (with reinforcement learning)	**79.5**

The results indicate that the application of reinforcement learning to our model significantly enhances its accuracy on the Visual Genome dataset, increasing it to 79.5% from 70.1% achieved without reinforcement learning. This improvement suggests that the reinforcement learning component is crucial for optimizing the model’s performance in complex image recognition tasks.

In this study, we introduced HybridBranchNetV2, a high-performing model that achieved exceptional results across various datasets. It achieved a remarkable 98.25% accuracy on the CIFAR-100 dataset and a 99.88% accuracy on the Flowers-102 dataset. Demonstrating its adaptability, our model was fine-tuned for the ImageNet dataset and reached an accuracy of 89.4%. Furthermore, HybridBranchNetV2 exhibited expertise in scene feature recognition, obtaining an mR@20 score of 28.3 on the Visual Genome dataset.

In ImageNet 5, we provide examples showcasing the capabilities of our network in the image content extraction phase.

- **Objects:** Man, Woman, Car, Road, Mountain, Sky, Tree, Surfboard, Sun, Guardrail, Shorts.
- **Relationships:** (man, in front of, car), (woman, behind, car), (car, on, roadside), (road, beside, mountain), (road, under, sky), (tree, under, sky), (man, facing, away), (woman, near, car door), (car, has, surfboard on top), (sun, shining on, scene), (man, wearing, shorts), (woman, standing, next to car), (car, facing, mountain), (mountain, under, sky), (man, near, guardrail), (guardrail, beside, road), (tree, beside, road), (surfboard, on top of, car), (mountain, in, background), (sky, above, everything).

Based on the results presented, it becomes evident that adding layer of textual and descriptive knowledge to each image has enhanced the network’s performance. This illustrates that deep neural networks possess significant learning potential when supplied with appropriate data and knowledge extraction techniques.

**Fig 4:**
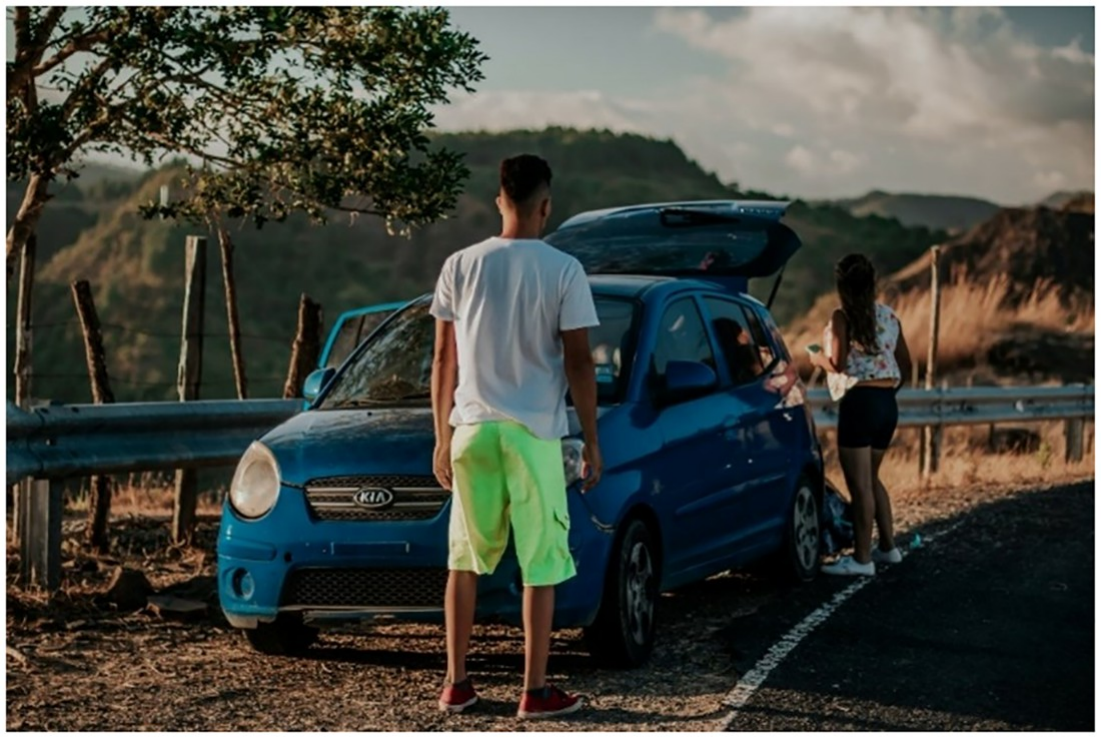
Exemplars of image content extraction using our network (Man standing in front of car near woman) [48].

**Fig 5:**
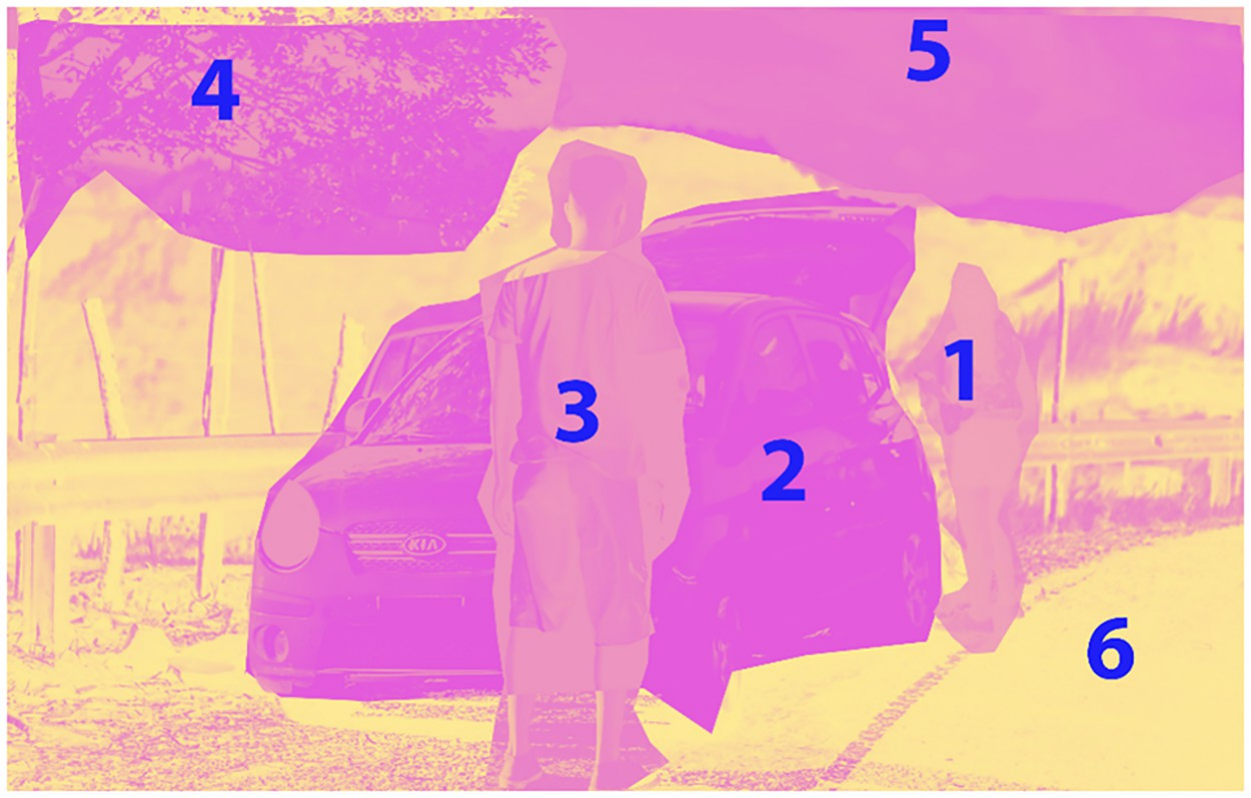
Exemplars of image content extraction using our network normalize heatmap.

Fig 4 presents a test image employed to examine the relationship extraction and corresponding heatmap of the proposed network [48]. In Fig 5, a normalized heatmap of the network prior to reinforcement learning layer is depicted. This heatmap illustrates how the activities associated with at least six objects in the image are distributed and enables the observation of each one separately. The image demonstrates the network’s capability to identify all objects present in the image. In the heatmap, all objects such as man, woman, car, tree, etc., exhibit uniform activation in feature extraction and are easily distinguishable, indicating that the relationship between objects in classification through graph utilization leads to the activation of specific regions of the image and decision-making. In existing classification algorithms, this possibility is usually absent, and only some objects are activated.

**Fig 6:**
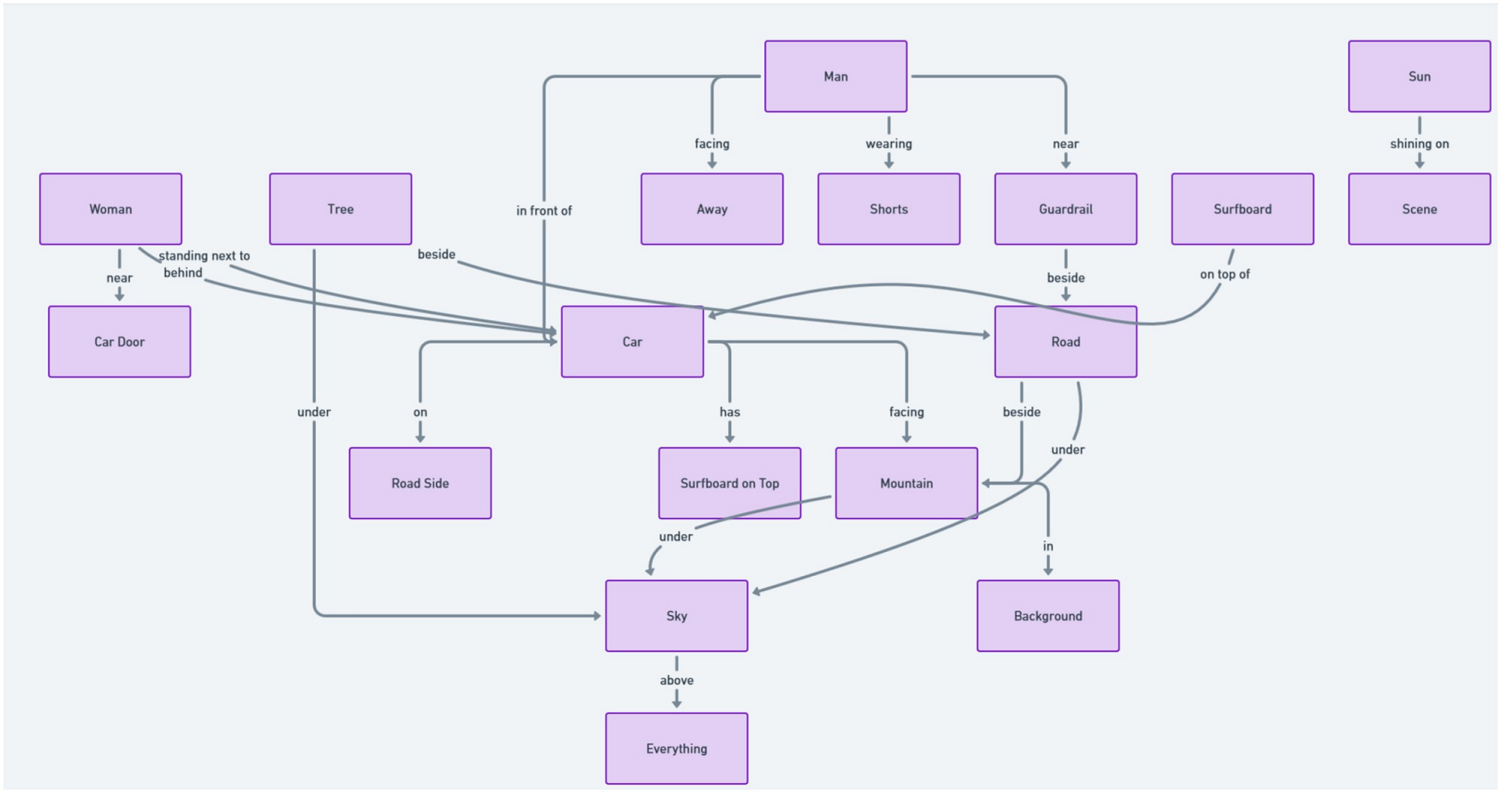
Relationships in a scene: Objects, people, and positions in the image provided in Fig 4.

The graph presented in Fig 6 visually captures the intricate relationships within a scene, portraying the dynamic interplay between various elements such as individuals, objects, and their spatial configurations. Each edge on the graph represents a specific relationship, whether it be the spatial positioning of an object in front of or behind another, the association between a person and a nearby entity, or the overarching connections that define the overall composition. The nodes, representing entities like people, cars, and natural elements, are strategically placed to highlight the contextual significance of their interactions.

This graphical representation, extracted from our proposed network, provides a concise and insightful overview of the complex network of relationships in the depicted scene. It provides a valuable visual aid for understanding the spatial dynamics and associations at play. This visualization not only simplifies the interpretation of complex relational structures but also helps identify key patterns and interactions within the scene. It enhances the network’s ability to analyze and comprehend the underlying complexities of the visual data.

### IV.IV. Performance comparison of various models on ImageNet-1k dataset

We compared the proposed model on the ImageNet 1K dataset with new deep learning models in terms of the number of model parameters, accuracy, FLOPs (number of operations per second), training time, and inference time and The result of this evaluation is shown in Table 8. The training and testing were conducted on a V100 system, ensuring consistent and reliable results. Additionally, the inference time was assessed on a V100 GPU with a batch size of 16, utilizing a codebase for fair and accurate comparison across different deep learning models. The CPU employed for these computations was a 50-core CPU.

**Table 8:** Comparison of deep learning models on ImageNet 1K dataset: Parameters, accuracy, and inference time.

Method	Top-1 Acc	FLOPs	#Params	Inference time(ms)	Training time (hours) (100 epochs)
HybridBranchNet3 [9]	83.1	19.3	9.12M	25	35
**HybridBranchNetV2**	**89.4**	33.4	16.5M	31	56
EfficientNet-B3 [42]	81.6	**1.9**	12M	43	66
EffNet-L2 [39]	88.3	36	121M	69	75
ResNet-101 [45]	83.0	13	48M	73	26
BotNet-T7-hybrid [49]	84.7	46	75M	105	145
RegNetY-16GF [50]	82.9	16	84M	96	130
EfficientNetV2-S [44]	83.9	8.8	22M	48	39
T2T-ViT-24 [51]	82.2	13	64M	59	120
DeiT-B-384 (ViT+reg) [52]	83.1	56	86M	60	120
Top-k DiffSortNets [53]	88.37	150	220M	93	-
Hiera-H [54]	86.9	125	673M	200	-

In Table 8, a comprehensive comparison is presented between the proposed model, HybridBranchNetV2, and several advanced deep learning models, such as EfficientNet, ResNet, and others, based on inference time, the number of parameters, and FLOPs. Despite having more parameters than its predecessor, HybridBranchNet3, the HybridBranchNetV2 model achieves an inference time of 31 milliseconds on a V100 GPU with a batch size of 16. This timing demonstrates the model’s capability for real-time applications, such as video stream analysis or use in edge devices, where maintaining low latency is essential. Additionally, experiments were conducted with smaller batch sizes, such as batch size 1, and results showed that the inference time remained below 40 milliseconds, which is well within the acceptable range for real-time image processing at frame rates of 25 to 30 FPS. The parameter efficiency of this model is particularly important for use in low-power devices or edge computing systems. With only 16.5 million parameters, HybridBranchNetV2 is significantly smaller than models like RegNetY (84M) or DeiT-B (86M). This characteristic makes it more suitable for devices with limited computational resources, such as embedded systems or mobile platforms. The smaller parameter size directly contributes to reduced memory consumption and lower computational overhead, which is critical in real-time systems. Moreover, quantizing the model allows for achieving higher speeds on edge hardware, although there may be a slight drop in accuracy. These features make the HybridBranchNetV2 model an attractive option for real-time applications and environments with limited resources.

The proposed method, in terms of model size and computational complexity, is slightly larger than HybridBranchNet3 and therefore operates a bit slower. However, it exhibits a 6.3% higher accuracy. It also has fewer parameters compared to models of similar complexity, which enables its deployment on low-power devices. Additionally, the proposed method does not necessitate extensive parameter tuning during training and testing, unlike the Top-k DiffSortNets approach. EfficientNet models are suitable for classification tasks. However, they are not capable of extracting knowledge among features of objects within an image. In contrast, our model uses the relationships between objects for training, which leads to better feature extraction. For this reason, the proposed method is more accurate compared to the EfficientNet models.

### IV.V. Evaluation of proposed method performance on ImageNet-Hard dataset

There exists a dataset called " ImageNet-Hard " consisting of 10,980 images from various collections such as ImageNet and its variants. This dataset is designed to challenge advanced machine vision models, focusing mainly on issues related to scale and spatial orientations in image classification. To evaluate the performance of the proposed method on the ImageNet-Hard dataset, we conducted tests to assess the generalization level of this approach and compare its accuracy with other methods.

**Fig 7:**
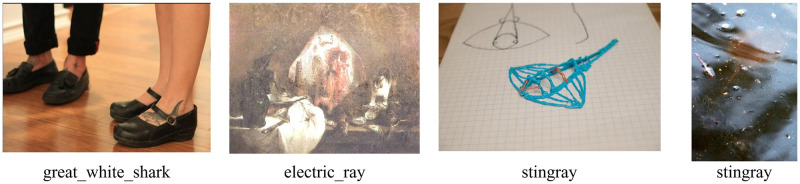
Selected examples from the ImageNet-Hard dataset.

The performance of models like CLIP-ViT-L/14 on this dataset has significantly deteriorated, with their accuracy reported to be very low. This indicates the complexities of the images in the dataset and the challenges associated with conditions where conventional imaging and processing methods cannot correctly identify essential details. This dataset is created to improve our understanding of the limitations of models and strengthen them by introducing them to more challenging conditions in image classification, conditions that deviate from the usual distribution. It aims to aid in enhancing image classification algorithms under conditions that are more difficult than normal. Several samples from the ImageNet-Hard dataset are depicted in Fig 7.

The accuracy of the proposed method on the ImageNet-Hard dataset is presented in Table 9.

**Table 9:** A comparison of the proposed method with image classification models on the ImageNet-Hard dataset.

Model [55]	Accuracy (%)
AlexNet [55]	7.34
VGG-16 [55]	12
ResNet-18 [55]	10.86
ResNet-50 [55]	14.74
ViT-B/32 [55]	18.52
EfficientNet-B0 [55]	16.57
EfficientNet-B7 [55]	23.2
CLIP-ViT-L/14@224 [55]	1.86
CLIP-ViT-L/14@336 [55]	2.02
Coca_ViT-L/14 [55]	**36.79**
HybridBranchNet3 [9]	14.5
HybridBranchNetV2	31.5

As found from Table 10, the proposed model, achieving an accuracy of 31.5%, notably demonstrates significant improvement compared to many other models. HybridBranchNetV2 employs more effective strategies to deal with the complexities of images in this dataset. These results can provide a better understanding of the limitations and capabilities of existing models under challenging conditions. As it is evident, the Coca outperforms the proposed method. The number of parameters in this model exceeds 2.1 billion, utilizing a strong language model. Whereas the proposed model has much fewer parameters (16.5 million) and does not utilize a language model.

**Table 10:** Comparison of model performance across multiple runs.

Model	Run 1	Run 2	Run 3	Run 4	Run 5	Run 6	Run 7	Run 8	Run 9	Run 10
HybridBranchNet3 [9]	85.20%	83.80%	82.50%	84.60%	82.10%	83.70%	83.90%	84.30%	84.80%	82.40%
HybridBranchNetV2	88.60%	89.10%	89.80%	90.20%	88.50%	88.90%	89.40%	88.70%	89.30%	90.10%

### IV.VI. Comparative analysis of Grad-CAM activation patterns in HybridBranchNetV2 and HybridBranchNet

**Fig 8:**
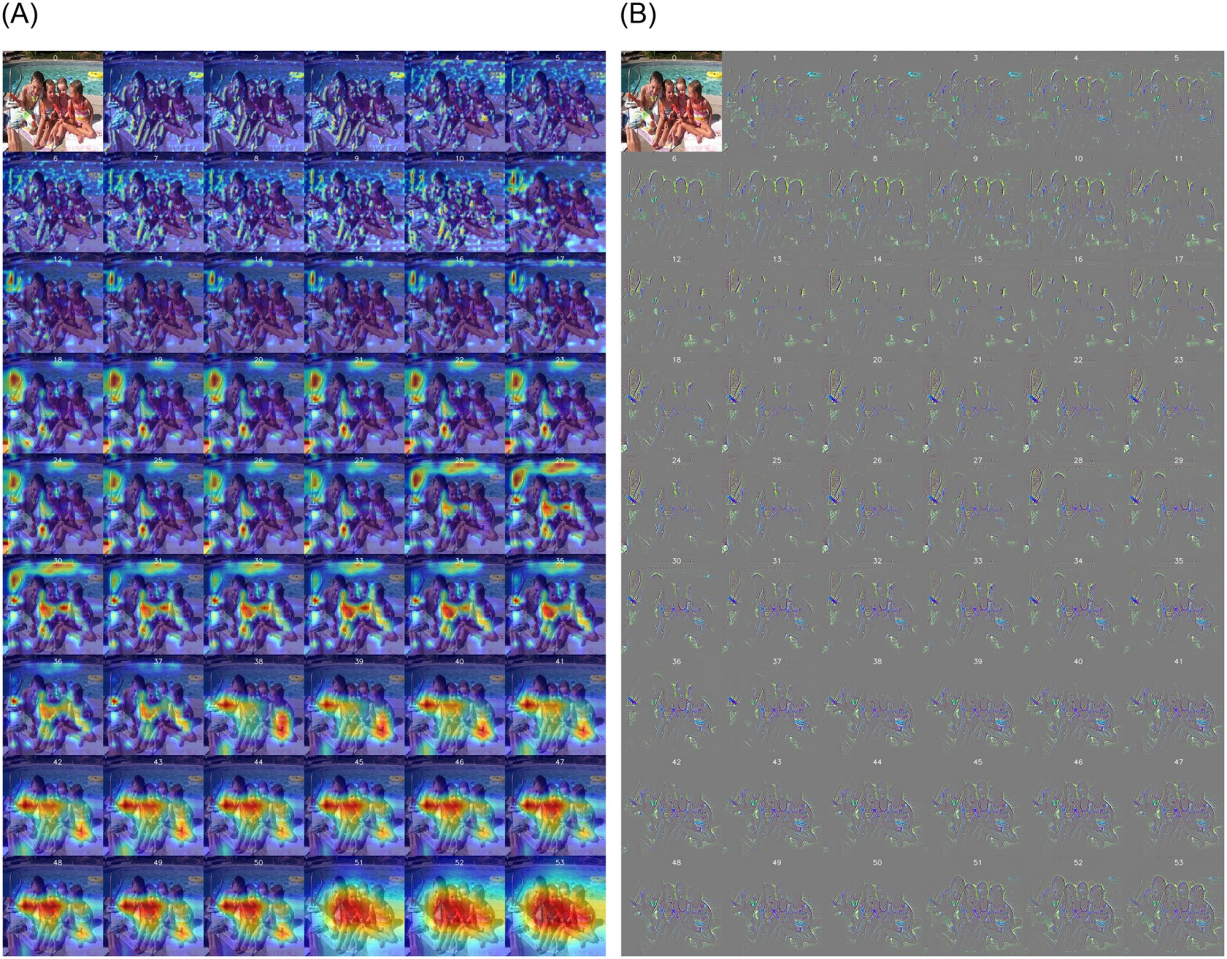
**A:** Grad-CAM with color in HybridBranchNetV2, **B:** Grad-CAM with feature in HybridBranchNetV2.

**Fig 9:**
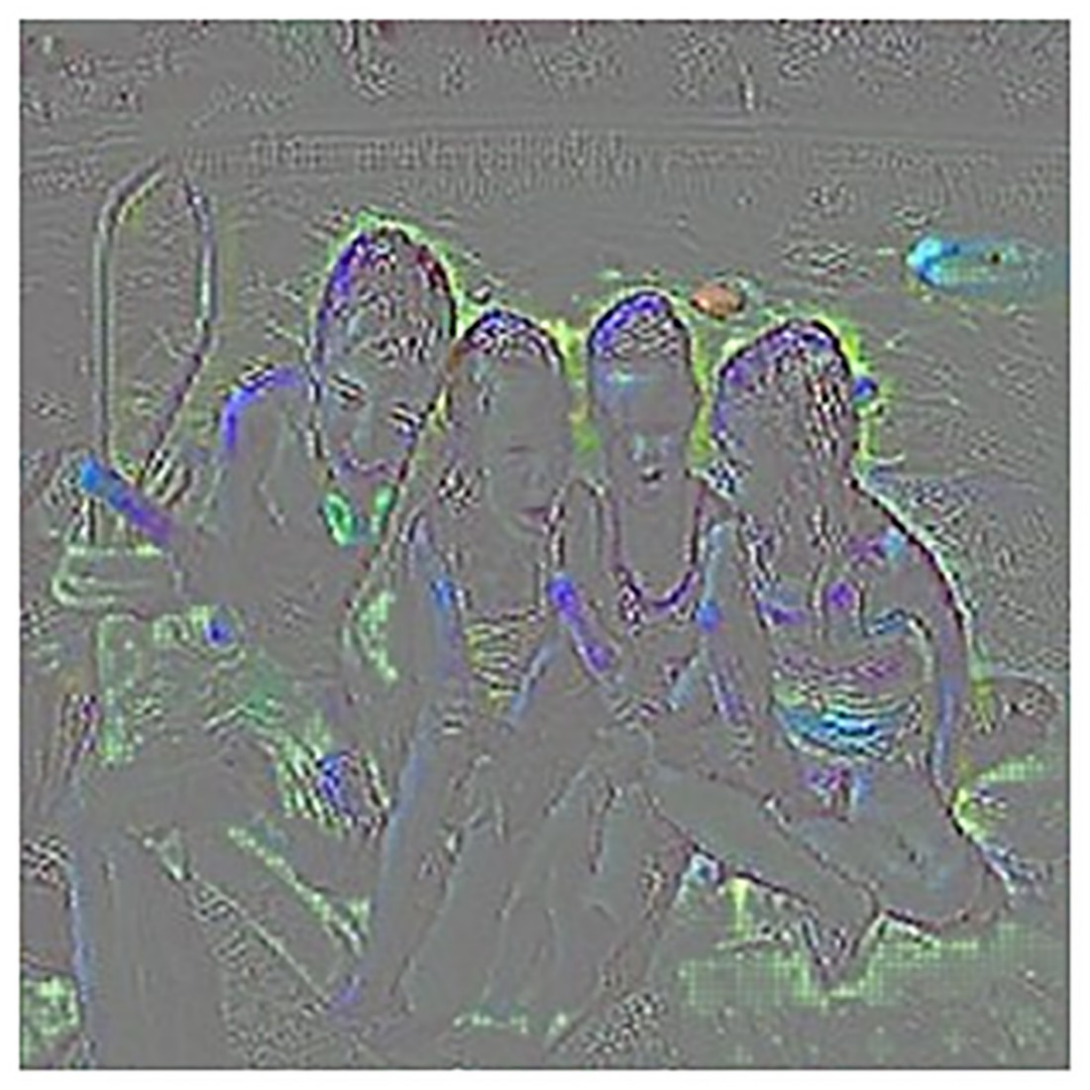
Last layers of Grad-CAM in HybridBranchNetV2.

**Fig 10:**
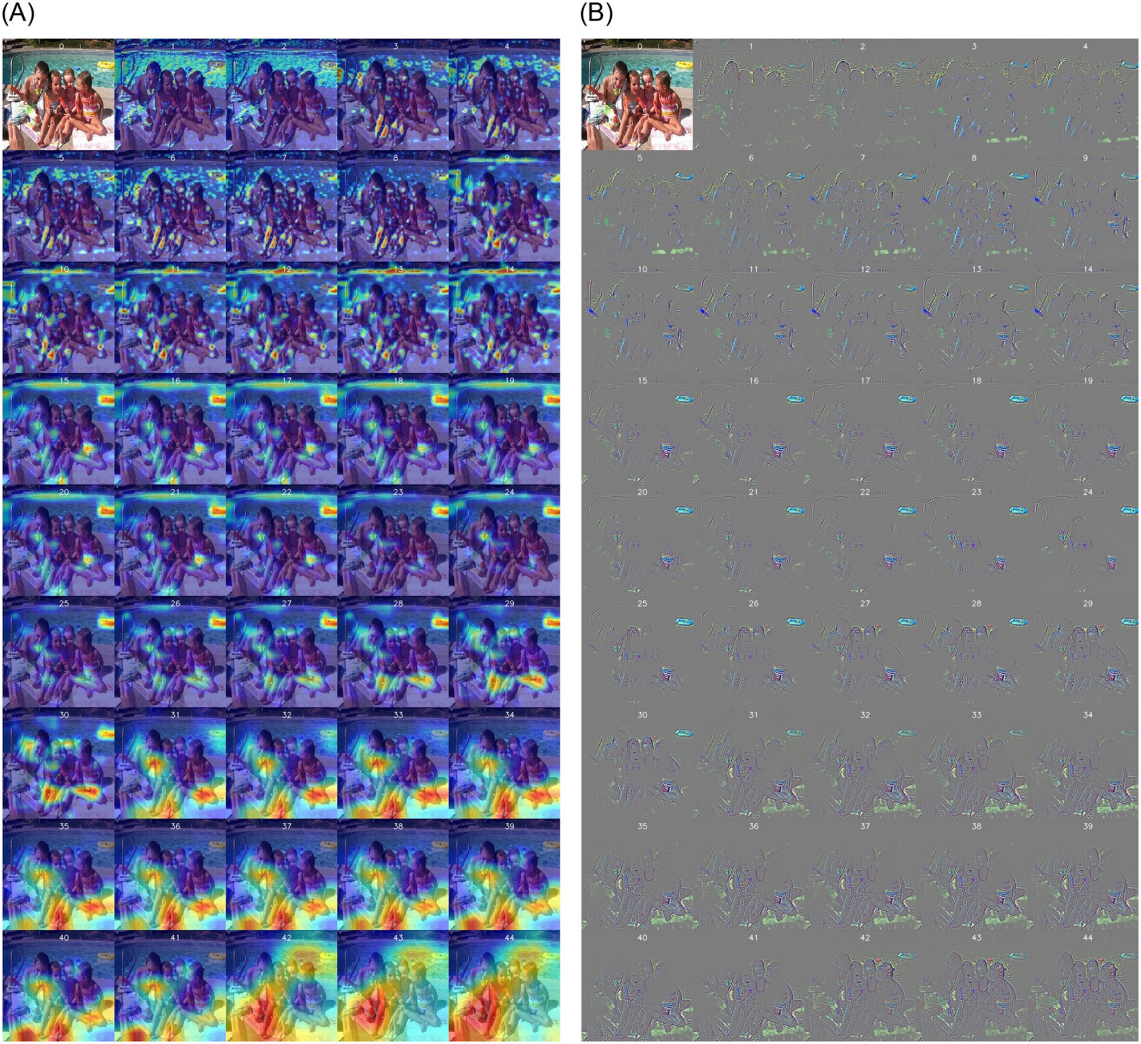
**A**: Grad-CAM with color–HybridBranchNet, **B:** Grad-CAM with feature–HybridBranchNet.

Comparing the feature maps of HybridBranchNetV2 and HybridBranchNet using Grad-CAM reveals distinct differences in their activation patterns. Grad-CAM [56] (Gradient-weighted Class Activation Mapping) is a technique used to visualize and understand the regions of an image that contribute the most to the final classification decision made by a convolutional neural network. In the proposed model, the convolutional layers used in the classification block are visualized using the Grad-CAM structure to understand which areas the network focuses on. The output displayed in Fig 8A indicates that the initial layers aim to extract general scene features and gradually focus on specific regions where the main object is located. This process demonstrates an increase in active regions in the network, indicating that the network correctly applies its attention mechanism and ultimately presents the desired object cohesively. In Fig 8B, considering the image details, undoubtedly, even the minutest details of the object are thoroughly extracted in the final analysis, rendering individuals distinctly recognizable. This property can be clearly inspected through Fig 9 which has been enlarged for clarity. Furthermore, in Fig 10, we illustrate the Grad-CAM output corresponding to the HybridBranchNet architecture. The differences between the two Grad-CAMs are observable, with the proposed model better and more accurately focusing on the target object.

The primary constraint of the proposed method is the requirement of a dataset that includes relationships between objects or provides a complete textual description of the presented image. In other words, the relationships between all objects and the relationship between each object and the background must be specified. Alternatively, the important shape features of objects must be precisely described. Based on our experience with HybridBranchNet model [9], we observed that improper feature extraction leads to some difficulties in the classification stage, specifically at the end of the convolutions. To elaborate further, we noticed that for some complex images, rich features were not correctly extracted when we use Grad-CAM. To address this issue, we decided to introduce a new method in this study, to provide more information about the image to the neural network and obtain richer extracted features. For this purpose, we added a component to the model that extracts relationships between objects and employed reinforcement learning (as described in Section III.V). This approach led to obtaining richer features consequently achieving a noticeable improvement in classification accuracy. This observation is confirmed by the Grad-CAM results in Fig 8 and the classification accuracy presented in Tables 4, 5 and 7.

### IV.VII. Comparative analysis of HybridBranchNet and HybridBranchNetV2 performance: A multirun study

To perform a comparative analysis of HybridBranchNet and HybridBranchNetV2 regarding their performance, we executed multiple runs of each model with varying random seeds, totaling 10 runs per model. Subsequently, we computed the variances of the evaluation metrics and determined the p-values. The performance metrics for each model across the 10 runs are presented in Table 10.

Upon analyzing the experimental results, we derived the following statistical insights:

For HybridBranchNet, variance of evaluation metrics is 2.15, and P-value is 0.032. For HybridBranchNetV2, variance of evaluation metrics is 0.54, and P-value is 0.001.

The outcomes indicate that HybridBranchNetV2 demonstrates significantly lower variance in its evaluation metrics compared to HybridBranchNet, as evidenced by both the variance values and the p-values. Consequently, HybridBranchNetV2 showcases more consistent performance across various random seeds, thereby establishing itself as the superior model in terms of stability.

Furthermore, in Table 11, a comparison between two methods, HybridBranchNetv2 and HybridBranchNet, is provided to facilitate a more comprehensive analysis.

**Table 11:** Comparison between two methods, HybridBranchNetv2 and HybridBranchNet.

Name	HybridBranchNetV2	HybridBranchNet
**Approach**	Utilizing textual knowledge along with visual features in deep neural networks.	Utilizing the nature of deep neural networks, extensive training samples, and specific feature extraction structures.
**Strengths**	1—Integration of linguistic and visual information for deeper comprehension 2- Improved classification accuracy 3- Efficient feature extraction without altering image resolution or increasing parameters 4- Incorporation of reinforcement learning for adaptive feature extraction 5- The need for fewer training parameters. 6—Extracting relationships between objects in the image.	1—Ease of use 2—applicability in various image-dependent scenarios 3- feature extraction based on adjustable network depth, width, and optimization. 4-Enhanced robustness against noisy or ambiguous input data, leading to more reliable classification outcomes.
**Weaknesses**	1—The complexity of model training at two different levels, before reinforcement learning and after, alongside parameter tuning for each. 2—The complexity of extracting the relationship matrix and filtering out unnecessary elements. 3- The need for datasets containing text or relationships between objects.	1—Challenges in independently grasping underlying concepts and semantic relationships between objects 2—Limited capacity to comprehend intricate concepts and relationships within images 3—Potential difficulties in handling large-scale datasets, leading to longer training times and increased computational resource requirements. 4—The risk of overfitting, especially when dealing with complex or noisy data, which can result in reduced generalization performance on unseen examples. 5—Dependency on high-quality labeled data for effective model training, which may not always be readily available or easily obtainable for all application domains.

### IV.VIII. Ethical management in AI architecture: Strategies and recommendations

In the proposed architecture, ethical issues can be managed through several different ways:

- Data Privacy Preservation:
  a. Employing local processing methods (such as fine-tuning) to train models using data not sent to cloud servers.
  b. Protecting individuals’ privacy by utilizing techniques like anonymization, removing personal information, and reducing the possibility of individual identification in the data.
- Reduction of Monopoly and Discrimination:
  a. Collecting data from diverse groups within the community to prevent bias in the model.
  b. Using techniques such as data augmentation to increase data diversity and reduce monopolistic practices.
- Social Impacts:
  a. Assessing the potential social impacts of the proposed models on society and cultures, considering their various applications, such as diagnosing skin diseases, object detection in images, etc.

Strategies and Recommendations:

- Extracting relationships between objects and training systems on data sets that prevent information disclosure and preserve privacy can ensure that the model does not operate on prohibited processes. In this way, training and extraction based on relationships between objects and specific features that the system possesses can help enhance privacy.

Implementing reinforcement learning models within the architecture to enforce boundaries and constraints, ensuring adherence to ethical, security, and other relevant criteria in the model’s outputs.

### IV.IX. Mitigating overfitting in model training: Strategies and empirical validation

To ensure that the model did not suffer from overfitting, we implemented several strategies:

- Regularization Techniques: We applied L2 regularization during training to penalize large weights and prevent the model from fitting noise in the training data. This helped control the model’s complexity and reduce overfitting.
- Cross-Validation: We utilized 5-fold cross-validation to evaluate the model’s performance. The consistent performance across different folds indicated that the model generalizes well to unseen data and is not overly fitted to the training set.
- Monitoring Training and Validation Loss: We plotted the training and validation loss curves during training. The convergence of both loss curves without a significant gap between them suggested that the model did not overfit the training data.
- Evaluation on Unseen Data: We evaluated the model on a separate test set that was not used during training. The similar performance between the test and validation sets indicated that the model generalized well to unseen data and did not memorize the training examples.
- Comparing Training and Test Performance: We observed that the model’s performance metrics on the training and test sets were comparable. This suggested that the model learned generalizable patterns from the training data without overfitting.
- Early Stopping: We implemented early stopping based on the validation loss. Training was halted when the validation loss stopped improving, preventing the model from overfitting to the training data.

By employing these strategies and observing empirical evidence such as consistent performance across different evaluation metrics, convergence of loss curves, and similar performance on unseen data, we ensured that the model did not suffer from overfitting.

### IV.X. Model misclassification analysis

**Fig 11:**
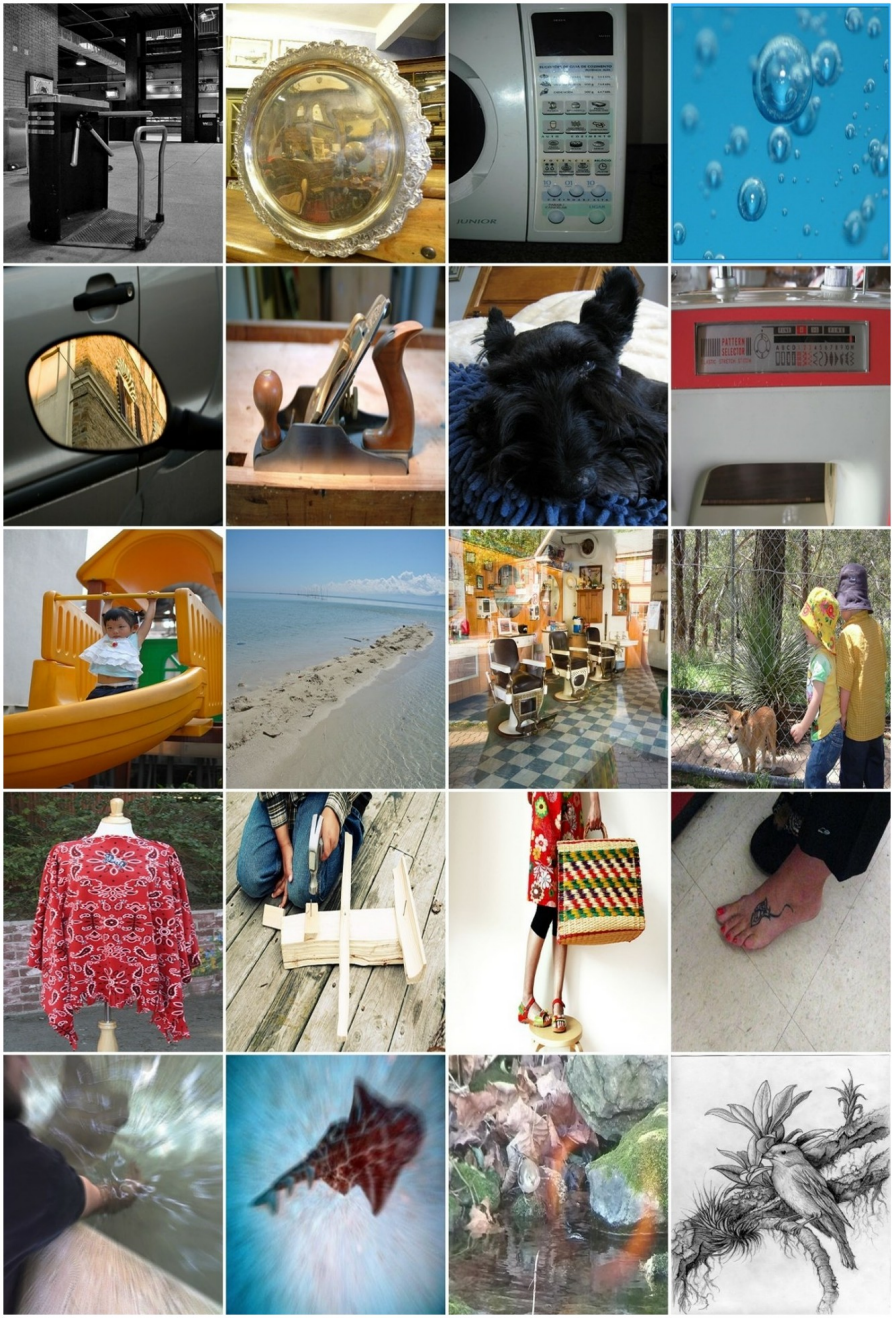
Analyzing misclassification in ImageNet 1K dataset: Insights from 20 randomly selected errors.

In this study, an analysis of the model’s errors was conducted, where 20 random samples of misclassifications were selected from the ImageNet 1K dataset in Fig 11. The examination of these samples revealed that the majority of classification errors occurred in complex environments containing multiple labeled classes within the dataset. In such scenarios, the model often encounters significant challenges in making accurate classifications.

The primary reason for these errors appears to stem from the presence of various features extracted from the objects within the images, which can lead to incorrect class selection by the model. Specifically, in these challenging contexts, the model struggles to differentiate between classes, resulting in the selection of unrelated categories. Although this issue should be mitigated by the use of a classification loss function, the observed outputs were not entirely unexpected. This suggests that the intricacies of the features and their relationships in multi-class settings can hinder the model’s ability to generalize effectively, ultimately impacting its classification performance. This finding underscores the importance of further refining feature extraction methods and exploring more sophisticated strategies for managing class overlap in complex environments to enhance the model’s accuracy and robustness.

## V. Discussion

The primary limitation of object classification in several crucial fields of machine vision is that it often acts merely as an algorithm for rich feature extraction. Object classification algorithms learn from extensive datasets, observing numerous categories with considerable diversity within each category. Due to the diverse training data, methods with high classification accuracy have broad applicability. Hence, presenting a classification method with high accuracy is essential. To increase classification accuracy, there is usually a need to increase the training parameters. However, augmenting training parameters in datasets with limited amounts does not necessarily yield satisfactory results. Therefore, to achieve greater generality of classification algorithms, it is necessary to reduce training parameters while maximizing accuracy. The goal of this article is to present a network that achieves high classification accuracy with an acceptable number of training parameters to ensure broader applicability across various tasks.

Through examinations of powerful previous methods like Coca and some transformer networks, it has been revealed that networks often gain knowledge beyond images. This knowledge can be linguistic, adding textual features to image features, thereby enhancing classification accuracy. However, incorporating a language model often leads to an increase in training parameters and introduces linguistic model errors into the feature extraction process. In the Coca model, adding a language model has created a model with approximately 2.1 billion parameters. To add knowledge beyond images to the proposed model, a low-cost method with low error is introduced, which will be discussed further.

The proposed method adds knowledge to our base model, i.e., HybridBranchNet. Upon examining the architecture of the HybridBranchNet method, it was evident that the features extracted by the final classification layers were not very rich, meaning they did not generate suitable heatmaps. Therefore, appropriate knowledge was added to the base structure, and in the proposed method, by considering the relationship between objects and reinforcing features in several last layers, the classification accuracy was improved. For this purpose, reinforcement learning operations were used to enhance features in the last layers, and by using graph architecture, object relationships in the network were considered. To account for relationships, a relational matrix was used. This relational matrix adds approximately 7.5 million parameters to the model, which is not significant, compared to the achieved accuracy increase. Additionally, the reinforcement learning applied does not add parameters to the network. Therefore, the proposed model requires only 16.5 million parameters to add knowledge, which is much less compared to the Coca method.

To demonstrate the generality of the proposed method, the model was trained on the Visual Genome dataset and tested on the ImageNet dataset, yielding good accuracy. Additionally, to demonstrate the performance and generality of the model, tests were conducted on smaller datasets in the field of image classification, resulting in higher accuracy compared to previous methods. Furthermore, to demonstrate the performance of the method, we went a step further and tested the model on the ImageNet-Hard dataset, indicating the power of the proposed method. Although the proposed method achieved lower accuracy than the Coca method in this dataset, the number of parameters in the proposed model is less than 0.01 of this method, demonstrating the value and efficiency of the proposed method.

## VI. Conclusion

In this article, a model with high accuracy and acceptable training parameters is presented. Previous powerful methods, such as Coca and some transformer networks, incorporate additional linguistic model-based knowledge into the network, which can increase classification accuracy. However, in these models, the number of parameters has significantly increased compared to the base version. Additionally, the linguistic model error has been transferred to the feature extraction part, ultimately leading to inadequate performance in some applications. The proposed method in this study, attempts to address the shortcomings of these approaches while requiring acceptable number of parameters, with no additional error to the feature extraction when embedding knowledge. The proposed model is considered an improved version of the HybridBranchNet method and is designed to be more accurate and practical in classification compared to the base model. Reinforcement learning features are utilized to provide a suitable policy for selecting and extracting new features. Additionally, the relational graph properties are exploited to specify object relationships in a given scene, aiding the network in finding the best class for each object and somewhat enhancing generalization. The structure of this model demonstrates that adding more information during neural network training can significantly increase image classification accuracy.

One limitation of the proposed model is its high dependence on object-scene relationships. The absence of a detailed description of object states in the dataset leads to errors in the training of the proposed model and will have inadequate generalization during testing. Therefore, in future research, we intend to incorporate suitable language models with fewer parameters and less semantic errors into the proposed model, and also improve reinforcement learning performance for faster and better convergence. Additionally, besides using language models, we plan to employ graph networks in the learning structure to extract and utilize graph features within deep neural networks. These future directions will help in further improving the accuracy, efficiency, and generalization capabilities of our model, making it more robust and versatile for various image classification tasks.
